# Heterogeneously integrated flexible microwave amplifiers on a cellulose nanofibril substrate

**DOI:** 10.1038/s41467-020-16957-4

**Published:** 2020-06-19

**Authors:** Huilong Zhang, Jinghao Li, Dong Liu, Seunghwan Min, Tzu-Hsuan Chang, Kanglin Xiong, Sung Hyun Park, Jisoo Kim, Yei Hwan Jung, Jeongpil Park, Juhwan Lee, Jung Han, Linda Katehi, Zhiyong Cai, Shaoqin Gong, Zhenqiang Ma

**Affiliations:** 1 Department of Electrical and Computer Engineering, University of Wisconsin–Madison, Madison, Wisconsin 53706 USA; 2 Wisconsin Institute for Discovery, University of Wisconsin–Madison, Madison, Wisconsin 53706 USA; 3 Forest Products Laboratory, USDA Forest Service, Madison, Wisconsin 53726 USA; 4 Department of Materials Science and Engineering, University of Wisconsin–Madison, Madison, Wisconsin 53706 USA; 5 Department of Electrical Engineering, National Taiwan University, Taipei, 10617 Taiwan; 6 Department of Electrical Engineering, Yale University, New Haven, Connecticut 06520 USA; 7 Department of Electrical and Computer Engineering, Texas A&M University, College Station, Texas 77843 USA; 8 Department of Biomedical Engineering, University of Wisconsin–Madison, Madison, Wisconsin 53706 USA

**Keywords:** Electrical and electronic engineering, Environmental, health and safety issues

## Abstract

Low-cost flexible microwave circuits with compact size and light weight are highly desirable for flexible wireless communication and other miniaturized microwave systems. However, the prevalent studies on flexible microwave electronics have only focused on individual flexible microwave elements such as transistors, inductors, capacitors, and transmission lines. Thinning down supporting substrate of rigid chip-based monolithic microwave integrated circuits has been the only approach toward flexible microwave integrated circuits. Here, we report a flexible microwave integrated circuit strategy integrating membrane AlGaN/GaN high electron mobility transistor with passive impedance matching networks on cellulose nanofibril paper. The strategy enables a heterogeneously integrated and, to our knowledge, the first flexible microwave amplifier that can output 10 mW power beyond 5 GHz and can also be easily disposed of due to the use of cellulose nanofibril paper as the circuit substrate. The demonstration represents a critical step forward in realizing flexible wireless communication devices.

## Abstract (web summary)

Though flexible microwave integrated circuits (MICs) are desirable for the construction of functional microwave amplifier circuits, realizing low cost III-V-based MMICs remains a challenge. Here, the authors report a heterogeneous integration strategy for the fabrication of flexible low-cost MICs.

## Introduction

Microwave circuits are essential components in modern wireless electronic devices, which generate, process, transmit, and receive signals beyond gigahertz (GHz). To date, microwave circuits have been mainly constructed on integrated semiconductor chips and/or on printed circuits boards (PCBs), both in rigid form. However, there are a number of existing and emerging applications that cannot be (or easily) addressed by the rigid form-based microwave electronics. For example, large-area (e.g., >several square meters) microwave systems (e.g., phased array systems) require individual chip packaging, module assembly, and inter-connections, thereby making such systems bulky and heavy weight with jeopardized operation reliability^1,2^. This renders the rigid-chip-based solution a very disadvantageous one. In addition, rigid-chip microwave electronics are also unable to address applications where light weight and low-cost electronic systems need to be squeezed in limited/irregular spaces such as small-footprint gadgets, e.g., wearable devices, drones, etc.

To address the existing and emerging microwave applications, the concept of flexible microwave electronics was initiated and has been explored for the last decade or so. Although individual flexible microwave active and discrete/distributed passive components^3–19^, and some simple flexible passive circuits^3,10,20^, have been demonstrated, using these components^3–19^ to construct a functional microwave amplifier circuit, one of the most important types of circuits in microwave systems, has never been reported. To date, the only reported approach to fabricating bendable/flexible microwave integrated circuits (MIC) is to thin down the substrates of rigid-chip-based monolithic MIC (MMIC)^21–23^ and yield flexible MMIC. The fabrication cost of this approach includes rigid-chip fabrication cost (substrate plus processing) and the thinning down cost (processing). Although the substrate cost of Si-based MMIC^22,23^ is a small portion of the total cost and thinning down the Si substrate to convert MMIC to flexible MMIC can be considered reasonably cost-effective, the same approach^21^ to fabricating III–V-based MMIC becomes cost-ineffective due to the high cost of III–V substrates and the epitaxy layers grown on them. Furthermore, substrate thinning down renders the chip brittle, increases handling challenges and possibly leads to limited yield, particularly for large-area chips^24^, which could further increase the final cost of a flexible chip.

When a thinned microwave chip is transferred to a microwave-compatible flexible substrate, heat dissipation of the active devices becomes a big concern, as the majority of available flexible substrates are known to have much lower thermal conductivities than semiconductor substrates. As a result, active devices that are robust against self-heating are much preferred over InP- and Si-based transistors^21–23^ for reliable operation. Furthermore, considering the environmental impact^10,25–27^ of flexible microwave electronics under the present situation of frequent upgrading/discarding of microwave systems-containing gadgets, environment-friendly flexible MIC (*f*MIC) that can be easily disposed of with the minimum amount of waste is highly desirable.

In this study, we present a novel heterogeneous integration approach to realizing III–V-based *f*MIC, where the simple fabrication procedures of III–V-based MMIC are inherited in the fabrication but without wasting expensive III–V substrates or epitaxial layers as opposed to MMIC substrate thinning, thus substantially reducing the materials cost. We chose to employ AlGaN/GaN high electron mobility transistor (HEMT) as the active device in this work considering its superior microwave properties^28^, extraordinary thermal reliability (see Supplementary Note 1 and Supplementary Table 1 for details) under high operating temperatures and environmental-friendly nature of GaN materials^29^. We further demonstrated the integration of the GaN-based *f*MIC on wood-derived cellulose nanofibril (CNF) substrate, which has been previously demonstrated to be microwave compatible and biodegradable^10^, and convenient disposal of the *f*MIC through incineration for easy adoption into mainstream municipal waste treatments^30^. As the first of its kind, the cost-effective GaN HEMT-based *f*MIC amplifier shows excellent small-signal and large-signal microwave performances even under severe mechanical bending.

## Results

### Strategies of *f*MIC fabrication

**Fig. 1:**
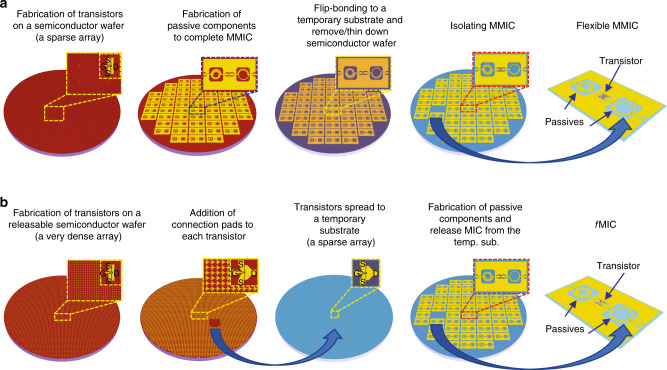
Two different strategies to realize flexible MIC. **a** Schematic illustration of transforming rigid MMIC to flexible MMIC via substrate removal. A sparse array of transistors was fabricated on a semiconductor epitaxial wafer. Passive microwave components were also fabricated on the same semiconductor wafer to obtain rigid MMIC. The wafer with MMICs was flip-bonded to a temporary substrate and the original semiconductor substrate was removed. After isolating MMICs, one MMIC was picked for use with or without a new host substrate. **b** *f*MIC based on heterogeneous integration of membrane transistors with passives on foreign host substrate. A dense array of transistors was fabricated on a semiconductor epitaxial wafer. The transistors are released from the semiconductor wafer after forming connection metal pads. Selected transistors were deterministically transfer printed on a reusable temporary substrate and formed a sparse array on it. Passive components were fabricated on the temporary substrate and together with the transistors to form MIC. *f*MIC is obtained after releasing the MIC from the temporary substrate.

Figure 1 presents schematic illustrations of two manufacturing processes that can be used for fabricating bendable MICs: flexible MMIC and *f*MIC. In the chip thinning down approach^21,31^ as shown in Fig. 1a, MMICs were built on III-V semiconductor epitaxial wafers that were thinned down to flexible form. The total number of MMICs that can be fabricated out of one epitaxial wafer using this approach is about (slightly smaller than) the area ratio between a wafer area (*A*_wafer_) and a circuit area (*A*_circuit_), which is similar to the turnout of previously demonstrated flexible electronics approaches^32–35^. For silicon-integrated circuits with a high density of transistors and for Si-substrate MMICs^22,23^, chip thin-down represents the most convenient approach to producing bendable chips and favorable cost advantages^36–38^, particularly considering the very low cost of Si substrates. However, for (majority of) MMIC chips built on epitaxial substrates (e.g., GaAs, InP, Sapphire, SiC, etc.), as the majority of the chip area is occupied by area-consuming passive components (e.g., inductors, capacitors, and transmission lines, etc.), the chip thin-down approach is no longer cost-effective due to the high cost of the epitaxial substrates.

The cost-effective approach to fabricate *f*MICs demonstrated herein, as shown in Fig. 1b, is to heterogeneously integrate membrane transistors^39,40^ transferred from a dense transistor-packed source epitaxial substrate, which have maximized the use of expensive source substrate, with passive components on an inexpensive, microwave-compatible destination substrate, thus avoiding the occupation of the source substrate by large-area passives. It is noted that the fabrication procedures and, therefore, the processing cost of both the active transistors and passive components are identical in the two approaches illustrated in Fig. 1. However, the substantial increase in the number of active transistors fabricated on the source substrate, now determined by the area ratio between *A*_wafer_ and the area of the transistors (*A*_transistors_), leads to the same number increase in fabricated *f*MIC (~13 times of *A*_wafer_/*A*_circuit_ as in Fig. 1a, detailed in Supplementary Note 2). It is also noted that the cost of the substrates of *f*MIC shown in Fig. 1b (CNF) is nearly negligible in comparison with that used in Fig. 1a. The only extra fabrication procedure used in Fig. 1b was transfer printing. As the transfer-printing techniques and the relevant tools have already been adopted for other mass-production applications^41,42^ and proven to be a low-cost addition in the applications, they also represent minimal cost additions in handling active transistors and passives upon adopting the present demonstration for massive production. As a result, the *f*MIC approach shown in Fig. 1b suggests substantial cost advantages.

**Fig. 2:**
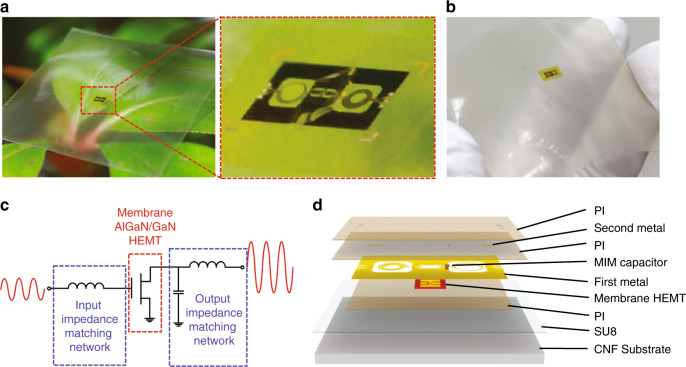
An *f*MIC amplifier on a CNF substrate. **a** A photograph of a fabricated *f*MIC amplifier on CNF substrate placed on a leaf. The magnified view shows image of the amplifier. **b** A photograph of flexible microwave amplifier under bent condition. **c** Schematic circuit diagram of the amplifier with denoted function of each part. **d** Exploded schematic layer view of *f*MIC amplifier on CNF substrate.

Figure 2a, b show photographs of a flexible microwave amplifier circuit on a transparent CNF substrate fabricated using the approach illustrated in Fig. 1b. The *f*MIC amplifier consists of AlGaN/GaN HEMT, which is readily available from large-size AlGaN/GaN-on-Si wafer^43^ and can be released in membrane form^13^. Figure 2c shows the circuit diagram of the flexible microwave amplifier. The designed operating frequency range of the amplifier is 5–6 GHz, which can address many commercial applications^44^. Due to the relatively long wavelength of electromagnetic (EM) wave within the operating frequency range, lumped spiral inductors and metal–insulator–metal (MIM) capacitors, instead of lengthy (at this frequency) transmission lines, were used to build compact passive impedance matching networks. Figure 2d shows the layer structure view and integration sequence of the amplifier circuit fabrication.

### Flexible AlGaN/GaN HEMT

Before constructing a flexible microwave amplifier on CNF substrates, we first fabricated and characterized membrane AlGaN/GaN HEMTs on the same CNF substrates. The purpose of the fabrication and characterizations is to extract the scattering (S-) parameters of the HEMT under the condition that the HEMT is implemented in the microwave amplifier. Supplementary Figs. 1 and 2 schematically illustrate the detailed fabrication process of membrane HEMTs on CNF substrates. The rationale for the choice of gate length and gate width of the HEMTs is explained in Supplementary Note 3. An array of HEMTs with a gate length of 300 nm (see Supplementary Figs. 3 and 4a) and a gate width of 90 μm was fabricated on an epitaxial AlGaN/GaN-on-Si wafer using conventional HEMT fabrication techniques. After completion of fabrications on the original rigid Si substrate, the HEMT array was diced into discrete HEMTs, each having an area of about 500 µm × 500 µm in size (see Supplementary Fig. 4b). It is noted that the HEMT size/area is still mainly dominated by the coplanar ground–signal–ground (G-S-G) probing pads needed for on-chip radio frequency (RF) characterizations. After dicing, an individual HEMT (or multiple HEMTs as needed) was picked up with a polydimethylsiloxane (PDMS) stamp after coating the HEMT with SiO_2_ by plasma-enhanced chemical vapor deposition (PECVD). The Si-handling substrate of the HEMT was then fully etched away using XeF_2_ etching (Supplementary Fig. 4c), leaving a membrane HEMT of about 3.5 μm-thick finished. The membrane HEMT on top of the PDMS stamp was then transfer printed to CNF substrates pre-coated with adhesion layers with the assistance of an MJB-3 contact aligner (Supplementary Fig. 5), of which the operation principle is the same as the commercial tools^41,42^. The images of HEMT sitting on CNF substrates are shown in Supplementary Fig. 6.

Direct current (DC) characteristics and S-parameters of the HEMT sitting on the original Si substrate and on CNF substrates were characterized, and the results are shown in Supplementary Figs. 7 and 8, respectively. Both drain current and peak transconductance were reduced after HEMT transfer to CNF substrates due to the increased self-heating effects in the device, which resulted from the low thermal conductivity of CNF substrate (~1 Wm^−1^ K^−1^)^45^ compared with that of Si substrate (~156 Wm^−1^ K^−1^)^46^. The results shown here are consistent with other reports^13,47,48^. Improving the thermal conductivity of flexible substrates^49,50^ is expected to improve the DC characteristics. Considering the low thermal conductivity of CNF and to avoid excessive heating, the HEMT was biased at a drain voltage (*V*_DS_) of only 10 V, despite HEMTs’ ability to sustain a much higher voltage (30 V)^51^.

**Fig. 3:**
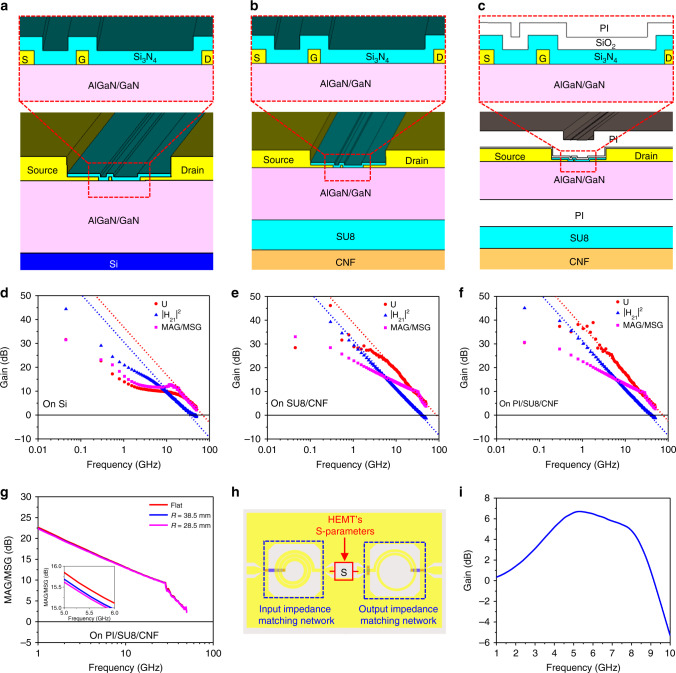
RF characteristics of membrane AlGaN/GaN HEMTs and *f*MIC amplifier design. **a**–**c** Cross-sectional schematic views of AlGaN/GaN HEMTs on Si (**a**), SU8/CNF (**b**), and PI/SU8/CNF (**c**) substrates. The magnified views in **a**–**c** show schematic views of the HEMTs’ active regions on different substrates. **d**–**f** Plots of measured values of current gain (|H_21_|^2^), Mason’s unilateral power gain (U), and maximum available gain/maximum stable gain (MAG/MSG) as a function of frequency of HEMTs on Si (**d**), SU8/CNF (**e**), and PI/SU8/CNF (**f**) substrates. **g** Plots of measured MAG/MSG values of HEMT on PI/SU8/CNF substrate as a function of frequency under flat and two bending radii: 38.5 mm and 28.5 mm). The inset is a detailed view of the MAG/MSG curves from 5 GHz to 6 GHz. **h** Layout of simulated *f*MIC amplifier. The amplifier simulations were performed by combining electromagnetic simulations of passive components with measured S-parameters of HEMT on PI/SU8/CNF substrate. **i** Simulated small-signal gain curve of the amplifier as a function of frequency.

The values of current gain (|H_21_|^2^), Mason’s unilateral power gain (U), and maximum available gain (MAG)/maximum stable gain (MSG) of the HEMTs were extracted from the measured S-parameters. The extracted values were plotted in Fig. 3d–f as a function of measurement frequency. Without de-embedding parasitic effects, the highest cut-off frequencies (*f*_T_) of ~34.1, ~37.9, and ~37.0 GHz, and the highest maximum oscillation frequencies (*f*_max_) of ~69.5, ~87.8, and ~75.0 GHz were obtained for HEMTs on Si, SU8/CNF, and polyimide (PI)/SU8/CNF substrates, respectively (Supplementary Table 2)^52^. The gate bias (*V*_GS_) values where the highest *f*_T_ and *f*_max_ were obtained were −1, −1.5, and −1 V, respectively, on the three different substrates. The RF performances of the HEMT on the CNF substrates are comparable to reported similar HEMTs on other substrates (see Supplementary Note 5 and Supplementary Table 3).

According to Eq. (1)^53^, it was expected that the HEMT on CNF substrate would have lower *f*_T_ than that on Si substrate due to the reduced peak transconductance (*g*_m_) on CNF. However, both *f*_T_ and *f*_max_ values on CNF substrates are higher than that on Si substrate. The reasons for the unexpected RF behavior were analyzed based on three-dimentional EM simulations using CST Microwave Studio^®^ (Supplementary Fig. 9a, b). The CNF substrates have lower dielectric constant (*ε*_r_) of ~2.6^54^ than Si (~8.9). The simulations show that the parasitic capacitance values (*C*_PAR_ in Eq. (1)) caused by the metal probing pads of the HEMT on CNF substrates were significantly reduced in comparison with that on Si substrates, which is consistent with other reports^13^, due to the lower *ε*_r_ of CNF (see Supplementary Fig. 9c–e and Supplementary Table 4). As no de-embedding of the parasitics were performed for the HEMTs, the parasitic effects were included in the measured (raw) HEMT S-parameters. The reduced parasitic effects of the HEMTs on CNF substrate have over-compensated for the adverse effects caused by the lower thermal conductivity of CNF substrate than Si on *f*_T_. As a result, higher *f*_T_ values were obtained for the HEMTs on CNF than on Si. The increase in *f*_max_ on CNF is primarily due to the increase in *f*_T_ and partly to the reduced gate-to-drain capacitance (*C*_GD_), which is also a consequence of the lower dielectric constant of CNF than Si as indicated in (2)^53^. As the HEMT on SU8/CNF does not have extra PI or oxide layers in comparison with PI/SU8/CNF as shown in Fig. 3b, c, the former has less extrinsic parasitic capacitances between electrodes and thus higher *f*_max_ values than the latter.

1 $$f_{\mathrm{{T}}} = g_{\mathrm{{m}}}/\left[ {2\pi \left( {C_{\mathrm{{G}}} + C_{\mathrm{{PAR}}}} \right)} \right]$$

2 $$f_{{\mathrm{max}}} \approx \sqrt {f_{\mathrm{{T}}}{\mathrm{/}}\left( {{\mathrm{8}}\pi R_{\mathrm{{G}}}C_{{\mathrm{GD}}}} \right)}$$

The HEMT on PI/SU8/CNF, which was used in amplifier circuit, was further tested under different mechanical bending conditions with bending radii (R) of 38.5 mm and 28.5 mm to study the influence of mechanical bending on the microwave performance of flexible HEMT (Fig. 3g). It was observed that the MAG of HEMT decreased very slightly in the interested frequency range as the bending radius became smaller (see the inset of Fig. 3g). The slight degradation of the small-signal power gain was due to the effects of external strain on the two-dimensional electron gas in the AlGaN/GaN heterostructure through piezoelectric effect and surface states, as revealed previously^55,56^. Correspondingly, the drain current^57^ and transconductance^58^ of the HEMT were slightly reduced, which in turn degrades its microwave performance.

### Design and fabrication of flexible microwave amplifier

The flexible microwave amplifier was designed using the characterized HEMT on PI/SU8/CNF, based on the circuit diagram shown in Fig. 2c. The passive components, two spiral inductors and one capacitor, consists of two layers of metal paths^10^. A layer of spin-cast PI isolates the two layers of metal path, which not only possesses good mechanical properties but also introduces low parasitic capacitances in the spiral inductors due to its low dielectric constant (~3.5). The optimized input impedance matching network contains a 2.5-turn spiral inductor connected in series with the input port. The optimized output impedance contains a low-pass inductor-capacitor network to suppress high-frequency harmonics, which is composed of a 1.5-turn spiral inductor connected in series with the output port and a MIM capacitor of 30 μm × 40 µm in size shunting the output of HEMT. The MIM capacitor employs a 140 nm-thick SiO_2_ layer deposited by electron-beam evaporation as its inter-metal dielectric layer. Figure 3h shows the circuit layout for circuit simulations, which combines S-parameters of the passive impedance matching networks obtained from the EM simulations and the measured S-parameters of the HEMT on PI/SU8/CNF. The simulation results shown in Fig. 3i indicate that the flexible microwave amplifier can achieve a peak small-signal gain of 6.69 dB at 5.3 GHz.

**Fig. 4:**
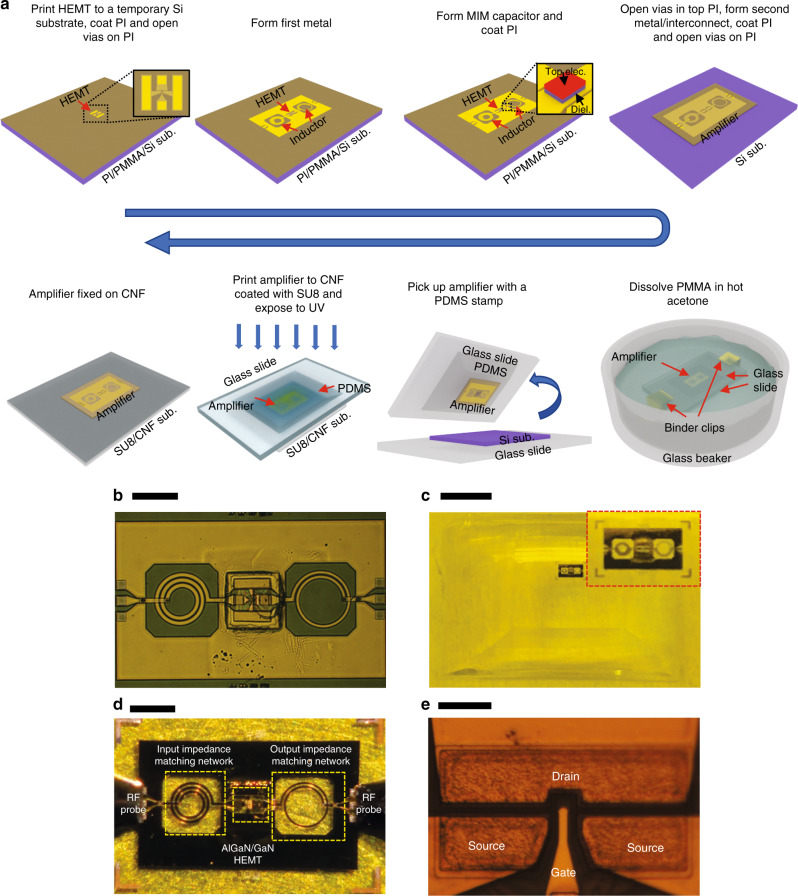
Fabrication process and images of *f*MIC amplifier on a CNF substrate. **a** Schematic illustration of fabrication process flow for amplifier on CNF substrate. A membrane AlGaN/GaN HEMT on PDMS stamp was transfer printed on a PI/PMMA/Si temporary substrate followed by PI encapsulation. After opening via holes on the PI, a first layer of metal was deposited and patterned to form the inductors’ spiral metal lines, the bottom electrode of MIM capacitor, and the ground plane. A stack of Ti/SiO_2_/Ti/Au was subsequently deposited to form the dielectric and top electrode of MIM capacitor, followed by casting of another layer of PI. After opening via holes on the PI at specific connection sites, a second layer of metal (for interconnect) was deposited and patterned. The completed membrane amplifier was picked up using a PDMS stamp after dissolving the sacrificial PMMA layer. The amplifier was then attached to a flexible CNF substrate with spin-cast SU8 as an adhesive layer. After UV light exposure for SU8 curing, the PDMS stamp was detached and the amplifier was fixed to the CNF substrate. **b** An optical microscope image of an amplifier on a temporary Si substrate. The scale bar is 0.4 mm. **c** A photograph of an amplifier on a PDMS stamp. The inset is a magnified view of the amplifier. The scale bar is 5 mm. **d** An optical microscope image of an amplifier on a CNF substrate under microwave measurements. The scale bar is 0.5 mm. **e** A magnified microscopic view of the active region of the membrane AlGaN/GaN HEMT that is integrated in the amplifier. The scale bar is 20 µm.

The amplifier fabrication process flow is shown in Fig. 4a. An extended schematic illustration of the process flow is shown in Supplementary Fig. 10. The amplifier fabrication began with picking up a rigid HEMT from a diced array of HEMTs (Supplementary Fig. 4b). The Si substrate of the HEMT was removed with XeF_2_ etching, leaving a membrane-form HEMT. The HEMT was then transfer printed to a temporary Si-handling substrate coated with a spin-cast PI layer and polymethylmethacrylate (PMMA) layer. The PMMA layer acts as a sacrificial layer and the PI as an adhesive layer. The input and output impedance matching networks were then fabricated on the Si temporary substrate. To minimize microwave propagation loss in the passive components, a thick metal stack (Ti/Cu/Ti/Au: 10/900/10/100 nm) was used for both layers of the metal paths of inductors, capacitor, and interconnects. A stack of Ti/SiO_2_/Ti/Au (10/140/10/100 nm) was deposited in one step to form the MIM dielectric layer and the top electrode layer of the MIM capacitors for a precise control of the capacitance value of the MIM capacitor^8^. Via holes through an insulation layer (PI: ~1.5 μm thick) were formed by reactive ion etching (RIE) and an interconnected metal was formed to connect the two layers of the metal paths, completing the fabrication of the amplifier on the temporary Si substrate. The PMMA sacrificial layer was then dissolved in hot acetone to release the amplifier (Si substrate can be reused) and the amplifier was transfer printed to a SU8-coasted CNF substrate to complete the entire fabrication process. As most of the above fabrication procedures were carried out on a rigid Si substrate, all the conventional MMIC fabrication techniques can be directly adopted here.

The uniqueness of the *f*MIC fabrication is that area-consuming passive components in the amplifier were integrated on an inexpensive CNF substrate and not on an epitaxial substrate (Fig. 1b). The fabrication method is drastically different from thinning down III–V-based rigid MMIC chip to obtain bendable MMIC^21,31^ (Fig. 1a). The *f*MIC method makes much greater use of epitaxial substrate materials and thus significantly reduces material cost.

As manufacturing microwave transistors requires high-cost fabrication processes such as electron-beam lithography, ohmic contact metal, and high-temperature annealing, the increase in active transistors’ density in the proposed approach leads to substantial reduction of processing cost per transistors as well, which is also the most important reason to employ larger semiconductor wafers in the semiconductor industry^59^. After transfer-printing membrane HEMT on temporary Si substrate, we fabricated the rest of the passive components using conventional low-cost photolithography systems with large feature sizes (e.g., at level of microns). The combined yet sensible use of high-cost processes for fabricating high density of transistors on epitaxial semiconductor wafer and low-cost processes for fabricating large-area passive components on temporary substrate at different *f*MIC fabrication stages enable a cheaper and faster fabrication process than conventional flexible MMIC approaches (see Supplementary Note 2 for detailed discussion). The conventional fabrication process and the reduced use of epitaxial materials together render the fabrication cost-effective. Only a very small piece of native epitaxial substrate was used for the HEMT fabrication, which has maximized the use of the epitaxial material.

Figure 4b–d show optical images of the amplifier before dissolving the PMMA sacrificial layer (on temporary Si substrate), after being picked up on PDMS stamp and sitting on CNF substrate during on-wafer microwave measurements, respectively. Figure 4e shows a microscopic image of the HEMT integrated in the amplifier. The flexible amplifier has a compact size of 1.4 mm × 2.4 mm, which is comparable to a rigid AlGaN/GaN HEMT-based MMIC chip^60^. As shown in Fig. 4d, 92.6% of the *f*MIC amplifier area was occupied by passive components and only the remaining 7.4% of the area was used by the HEMT, for which the G-S-G pads of the HEMT still account for the majority HEMT area (see Supplementary Fig. 4).

### Small-signal characterizations of flexible microwave amplifier

**Fig. 5:**
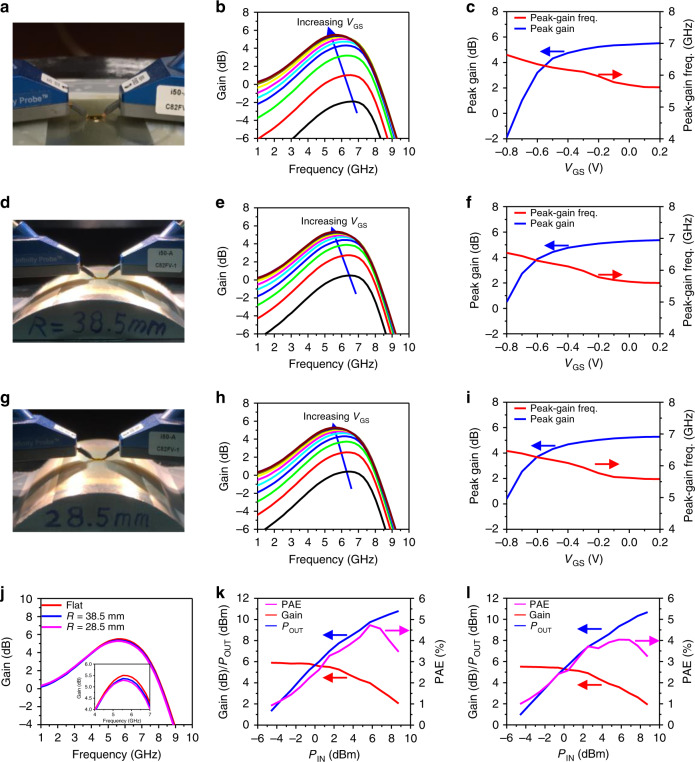
Microwave characterizations of amplifier on CNF substrate. **a**, **d**, **g** Photographs of an amplifier during microwave characterizations under flat (**a**) and bent conditions (**d**, *R* = 38.5 mm; **g**, *R* = 28.5 mm). **b**, **e**, **h** Measured small-signal gain values of the amplifier as a function of frequency under flat (**b**) and bent conditions (**e**, *R* = 38.5 mm; **h**, *R* = 28.5 mm). During the measurements, *V*_DS_ of the HEMT in the amplifier was set at 10 V and *V*_GS_ was swept from −0.8 V to 0.2 V with a step size of 0.1 V. **c**, **f**, **i** Plots of peak small-signal gain values and the corresponding frequencies as a function of *V*_GS_ of the amplifier under flat (**c**) and bent conditions (**f**, *R* = 38.5 mm; **i**, *R* = 28.5 mm). **j** Comparison of the measured peak small-signal gain values (i.e., under *V*_DS_ = 10 V and *V*_GS_ = 0.2 V) under flat and bent conditions. The inset shows the magnified view of the curves from 5 GHz to 6 GHz. **k**, **l** Measured *P*_OUT_, Gain, and PAE as functions of *P*_IN_ of the amplifier under flat (**k**) and bent condition (**i**, *R* = 38.5 mm) under continuous-wave operation mode.

The small-signal microwave performances of the amplifier were characterized on flat (Fig. 5a) and bent surfaces with bending radii of *R* = 38.5 mm (Fig. 5d) and *R* = 28.5 mm (Fig. 5g). Figure 5b, e, h present the measured small-signal gain as a function of frequency under flat and bent radii of 38.5 mm and 28.5 mm, respectively. To measure the small-signal gain values, the HEMT in the amplifier was biased at 10 V for *V*_DS_ and the *V*_GS_ was swept from −0.8 V to 0.2 V with a 0.1 V step. As the *V*_GS_ was increased, the small-signal gain values increased until peak-gain values were reached at 5.51, 5.37, and 5.29 dB at *V*_GS_ of 0.2 V under the three mechanical conditions. It was noted that the peak-gain frequency under the three conditions also shifted to lower frequencies (5.62, 5.60, and 5.58 GHz, respectively) as the bias was increased. To clearly illustrate the trend of the peak-gain shift, Fig. 5c, f, i plot the extracted peak-gain values and the peak-gain frequencies from Fig. 5b, e, h, respectively, as a function of *V*_GS_. As the *V*_GS_ increased from −0.8 V to 0.2 V, the monitored drain current of the HEMT in the *f*MIC also increased (Supplementary Fig. 11) and so did the transconductance of the HEMT (Supplementary Fig. 7b), which led to the increase in the RF gain values of the HEMT (Eq. (1)) and the small-signal power gain of the amplifier. Shifting of the peak-gain frequency was also observed. The maximum small-signal gain curves obtained at *V*_GS_ of 0.2 V under flat and bent conditions were compared in Fig. 5j. Overall, the small-signal gain changes were minimal due to bending, showing the mechanical bendability and robustness of the amplifier against bending. Supplementary Table 5 summarizes simulated and measured gain values of the amplifier at the interested frequencies and peak-gain frequency shifting values.

The slight degradation of the small-signal power gain of the amplifier and the observed shifting of the peak-gain frequencies when the amplifier was bent were a result of the degraded RF performance of the HEMT in the amplifier (see Fig. 3g), as well as by the slight impedance mismatch between the input/output matching networks and HEMT in the amplifier circuit. The input and output impedance values of the HEMT could be slightly changed as its bias was changed. Previous studies^6^ have also shown that inductance value of spiral inductors and capacitance value of the MIM capacitor can be altered under certain degrees of mechanical deformation. These changes led to the shifting of impedance matching in the amplifier and thus gain degradation, which was qualitatively verified by simulations (see Supplementary Note 6, Supplementary Table 6, and Supplementary Figs. 12 and 13 for details). According to the reported trend^55,56^, it is expected that as the bending radius of the amplifier is further increased, the performance of the HEMT and therefore the amplifier performance will continue to degrade until a mechanical failure occurs when the limit of the tensile strain of GaN is reached.

### Large-signal characterizations of flexible microwave amplifier

Although the amplifier circuit was designed using the small-signal S-parameters of the HEMT, large-signal microwave characteristics of the amplifier circuit were characterized to estimate the RF power handling capabilities of the *f*MIC for continuous-wave mode operation. Supplementary Fig. 14 shows the measurement setup for large-signal microwave characterization of the amplifier. The microwave loss in cables, bias tee, and connectors in the measurement setup was calibrated. A microwave signal generator was used to sweep the input power level at 5.5 GHz, the designed center frequency. The initial bias of the HEMT in the amplifier was fixed at *V*_GS_ of 0.2 V and *V*_DS_ of 10 V. Figure 5k (flat) plots the output microwave power (*P*_OUT_), the large-signal power gain (Gain), and the power-added efficiency (PAE) of the amplifier as a function of input microwave power (*P*_IN_). Figure 5k shows that the amplifier can achieve a peak PAE of 4.7% at *P*_IN_ of 5.76 dBm without bending. The corresponding *P*_OUT_ at the peak PAE was 9.75 dBm (9.44 mW) and the concurrent gain was 3.99 dB with a gain compression of 2.25 dB from the small-signal gain of 6.24 dB at 5.5 GHz. The *P*_OUT_ at −1 dB gain compression point (*P*_OUT_ | _−1 dB_) was 7.76 dBm (5.97 mW) and *P*_OUT_ | _−3 dB_ was 10.3 dBm (i.e., 10.64 ± 0.19 mW). To our knowledge, this is the first report of a flexible microwave amplifier that can output microwave power larger than 10 mW beyond 5 GHz. Supplementary Table 7 compares the various parameters of the amplifier with that of a SiGe BiCMOS power amplifier on thinned Si substrate^23^, illustrating the various performance advantages of the HEMT *f*MIC amplifier.

The large-signal microwave performance of the amplifier under mechanical bending (*R* = 38.5 mm, Fig. 5d) was further characterized and the results are shown in Fig. 5l. A peak PAE of 4.04% was measured along with *P*_OUT_ of 9.35 dBm (8.61 mW) and gain of 3.59 dB. The *P*_OUT_ | _−1 dB_ and *P*_OUT_ | _−3 dB_ were 7.98 dBm (6.28 mW) and 9.85 dBm (9.66 mW), respectively. The slightly larger *P*_OUT_ | _−1 dB_ under bending condition compared with the flat condition was due to a larger *P*_IN_. These results indicated that the amplifier is mechanically reliable for microwave power amplification.

Furthermore, based on the measured parameters shown above, the temperature distribution of the HEMT in the amplifier was analyzed using simulations. The detailed simulations are shown in Supplementary Fig. 15. The highest temperature of the HEMT experienced during large-signal operation was estimated to be around 184 °C. According to the reported thermal reliability of GaN HEMTs (Supplementary Table 1), the amplifier is also considered thermally reliable.

### Disposal of flexible microwave amplifier

**Fig. 6:**
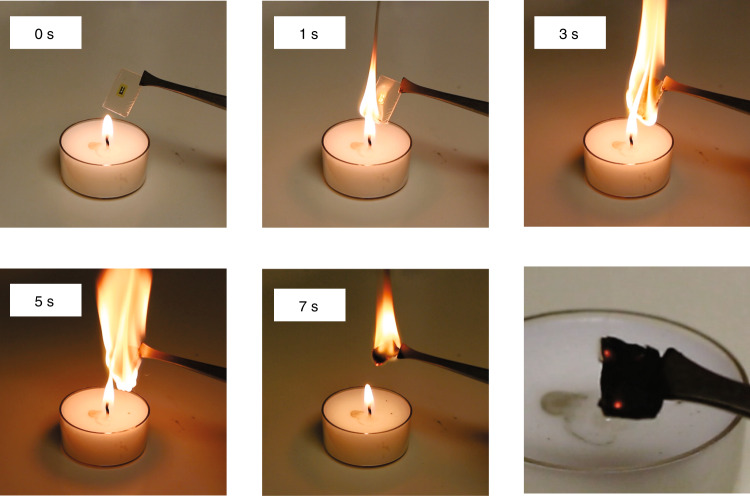
Disposal of microwave amplifier. Photographs of an amplifier at different stages of incineration. The disposal of the amplifier was complete in 7 s. The photograph at the bottom right shows the residue of the amplifier after incineration.

Although rigid-chip- and the rigid-board-based MICs are not easily disposed of and prone to electronic waste, flexible CNF substrate-based electronics have been shown to be biodegradable^10,61^, a significant benefit over other flexible host substrates such as polyethylene terephthalate and PI. In the demonstrated amplifier, a simple calculation showed that the CNF substrate accounted for ~92% weight of the amplifier. As an alternative approach to biodegradation via fungi^10^ to the disposal of the CNF-based *f*MIC circuit, disposition via incineration was attempted. As shown in Fig. 6, the CNF-based amplifier burst into flames instantly when ignited by a candle and turned into ashes after ~7 s, which is similar to that of other wood-based devices^62,63^. Although a detailed analysis was unable to be performed during incineration, it is expected that the major composition of the gas produced was carbon dioxide considering the highest possible temperature (>1100 K) of a candle flame^64^. The easy disposal of the amplifier indicates the green feature of *f*MIC approach, which could be environmentally significant if such *f*MIC is massively produced in the future.

## Discussion

In summary, we have demonstrated a viable approach to producing cost-effective, easy-to-implement, green, flexible microwave amplification circuits that may address many microwave/wireless applications. With a minimal addition of a new tool for transfer printing, the demonstrated approach is ready for adoption into large-scale manufacturing. Despite that GaN HEMT was specifically used in the demonstration, the approach can be applied to other semiconductor materials as long as the same principles apply. It is noted that further reduction of the active transistor size by minimizing their connecting pads is readily feasible. Research on flexible amplifiers toward higher power and/or higher frequency, which requires overcoming greater challenges of heat dissipation and reduction of parasitics than what is revealed herein and on other transistor-containing microwave circuits represents important directions of explorations in the future.

## Methods

### Preparation of CNF substrate

The tetramethylpiperidine-1-oxy-oxidized CNFs suspension was mixed at 0.9% weight percent using a mechanical mixer (Brookfield, Model L692) and further homogenized by sonication (Fisher Scientific, Model CL-334). Subsequently, the suspension was filtered with a hydrophilic polytetrafluoroethylene membrane (pore size: 0.1 μm, Omnipore membrane filter, model JVWP14225, Millipore Corporation, USA) under air pressure (1 MPa). Following filtration, wet CNF films were placed between a filter paper and a metal plate for further drying with a 10 kg weight on top of metal plate to achieve a flat and smooth surface for the CNF paper. The dried CNF paper was coated with bio-based epoxy resin (SUPER SAP® ONE, Hayward, CA) and sandwiched between two vacuum bagging films under vacuum for 24 h to cure.

### Fabrication of AlGaN/GaN HEMT array on Si and membrane HEMT

The AlGaN/GaN heterostructure was grown on a 1 mm-thick Si (111) substrate using a metal organic chemical vapor deposition system. The detailed AlGaN/GaN HEMT layer structure is illustrated in Supplementary Fig. 1. The fabrication of HEMTs began with deposition of source and drain metals (Ti/Al/Ni/Au: 20/200/45/55 nm), followed by a rapid thermal anneal at 800 °C for 90 s in nitrogen ambient to form ohmic contacts. The active region was defined by etching ~90 nm AlGaN/GaN layer using inductively coupled-RIE (Plasma-Therm SLR 770, BCl_3_/Cl_2_: 20/20 s.c.c.m., pressure: 5 mTorr, plasma power: 100 W, and inductor power: 300 W). Gate electrodes were defined using electron-beam lithography (NPGS on Zeiss (LEO) 1530 Scanning Electron Microscope) and Ni/Au (15/100 nm) Schottky gate contact. About 170 nm SiN_*x*_ was deposited using PECVD to passivate the surface of the AlGaN/GaN HEMT (Plasma-Therm PT70, N_2_/2% SiH_4_/5% NH_3_: 750/83.3/200 s.c.c.m., pressure: 400 mTorr, RF power: 100 W and *T*: 250 °C). Then, via holes on top of the electrodes were formed using RIE (Unaxis 790, CF_4_/O_2_: 45/5 s.c.c.m., pressure: 40 mTorr, and RF power: 100 W, ibid.). Finally, Ti/Cu/Ti/Au (10/900/10/100 nm) was used to form G-S-G RF metal pads. The HEMTs in the fabricated array were separated with a mechanical dicing saw, followed by deposition of SiO_2_ of ~150 nm-thick using PECVD (Plasma-Therm PT70, N_2_O/2% SiH_4_: 900/400 s.c.c.m., pressure: 900 mTorr, RF power: 25 W, and *T*: 250 °C). A selected HEMT was picked up using a PDMS (Slygard 184, Dow Corning, pre-polymer: curing agent = 10 : 1) stamp with the original Si substrate side exposed. The Si substrate of HEMT was fully etched using XeF_2_ dry etching (Xactix^®^ e2 XeF_2_ Etcher, 15 cycles, XeF_2_ pressure: 1.8 Torr, cycle duration: 120 s) and membrane HEMT was obtained on PDMS stamp (see schematic illustration in Supplementary Fig. 1).

### Fabrication of HEMT on CNF substrates

To fabricate HEMT on SU8/CNF, the membrane HEMT on PDMS stamp was printed to a CNF substrate that was spin-cast SU8 as an adhesive layer (SU8 2002, 4000 r.p.m., 30 s) using a mask aligner (MJB-3, Karl Suss) (Supplementary Fig. 5). After 5 min UV exposure (5.5 W/cm^2^) to cure the SU8, the PDMS stamp was retracted and the membrane HEMT was fixed on the CNF substrate (see Supplementary Fig. 2a). To characterize the HEMT, the PECVD oxide protection layer can be stripped off using a buffered oxide etchant (BOE, 6 : 1).

To fabricate HEMT on PI/SU8/CNF, a temporary Si substrate was prepared by spin-coating a layer of PMMA (PMMA 950 A2, 3000 r.p.m. 30 s, baked at 180 °C for 3 min) as a sacrificial layer. Then PI was spin-cast (4000 r.p.m. 30 s) on the Si substrate twice and baked at 150 °C for 4 min each time, followed by baking at 200 °C for 30 min and 350 °C for 3 h in nitrogen ambient (ibid). Then the PI was spin-cast (3000 r.p.m. 30 s) again and baked at 100 °C for 45 s (partially cured), followed by contacting the membrane HEMT on a PDMS stamp with the partially cured PI. The membrane HEMT was printed on the PI/PMMA/Si substrate after 1 min by detaching the PDMS stamp followed by baking at 150 °C for 4 min. The HEMT was then encapsulated by spin-casting a layer of PI on the HEMT. Using a layer of 100 nm-thick Cu as a hard mask, via holes were formed on the electrodes of HEMT using plasma to consecutively etch of PI (Unaxis 790, CF_4_/O_2_: 2/80 s.c.c.m., pressure: 75 mTorr, RF power: 100 W) and oxide (Unaxis 790, CF_4_/O_2_: 45/5 s.c.c.m, pressure: 40 mTorr, RF power: 100 W). After removing the Cu layer (Copper Etchant APS-100), the HEMT sitting on the PI/PMMA/Si structure was immersed in hot acetone (baked on hotplate at 200 °C) for 2 h, to remove the PMMA layer and release the membrane HEMT (encapsulated in two PI layers) from the Si substrate. Finally, the membrane HEMT was printed on a CNF substrate coated with SU8, followed by UV curing (see Supplementary Fig. 2b).

### Fabrication of amplifier on CNF substrates

The fabrication process of amplifier consists of HEMT fabrication and passives fabrication. The HEMT fabrication completely resembled the procedures that were described above. After the via holes were opened on the electrodes of HEMT sitting a PI/PMMA/Si-handling substrate, a Ti/Cu/Ti/Au stack layer (10/900/10/100 nm) was deposited to form a ground plane, spiral metal lines of inductors, and bottom electrode of MIM capacitor. Next, Ti/SiO_2_ (10/140 nm) and Ti/Au (10/100 nm) were deposited as the dielectric layer and top electrode of the MIM capacitor, respectively. A PI layer was spin-cast and cured as an insulation layer. Via holes through the PI layer were formed using RIE (Unaxis 790) with a copper hard mask (100 nm). Ti/Cu/Ti/Au (10/900/10/100 nm) was deposited as interconnect metal, followed by PI encapsulation. Via holes were formed to expose input and output coplanar G-S-G pads using RIE (Unaxis 790). The amplifier was released in hot acetone and then transfer printed to a SU8-coated CNF substrate (Fig. 4a and Supplementary Fig. 10).

### RF measurements and analysis

Semiconductor parameter analyzers (HP 4155B and Keithley 4200-SCS) were used to measure DC characteristics of AlGaN/GaN HEMTs on different substrates. S-parameters of the HEMTs on different host substrates and that of the amplifier on CNF under different bending conditions were measured using a vector network analyzer (Agilent PNA E8364A), after finishing standard on-chip calibration using a short-open-load-thru (SOLT) calibration kit. The DC bias voltages were supplied by the HP 4155B. Large-signal continuous-wave microwave characteristics of the amplifier were measured using the measurement setup shown in Supplementary Fig. 14. The 5.5 GHz signal was generated using a microwave signal generator (HP 83592B). A DC power supply (Agilent E3631A) was used to provide DC biases for the amplifier through its input/output ports using bias tees (HP 11590B). G-S-G microwave probes (Infinity Probe, Cascade Microtech) were used for on-chip probing. The output RF signal was first attenuated with a microwave attenuator (MOD 20600-6, Omni Spectra), then sensed by a power sensor (HP 8481 A), and finally was read with a microwave power meter (Agilent EPM-442A).

EM properties of the metal pads on Si and CNF structures were simulated using CST microwave studio^®^. The simulations of the small-signal microwave performance of the amplifier under flat and bent conditions performed through EM/circuit co-simulations of passive impedance matching networks and the measured S-parameters of the HEMT on PI/SU8/CNF substrates using Advanced Design System (Keysight).

### Thermal analysis of HEMT in amplifier

Finite element analysis was conducted using COMSOL Multiphysics^®^ (Supplementary Fig. 15). The copper thickness of 1 µm (thermal conductivity, *K* = 380 W/m·K) was set as metal ground plane and connected to 500 µm × 500 µm × 3.5 µm (thickness) GaN (*K* = 130 W/m·K). The HEMT was encapsulated in 12 µm thick PI (*κ* = 0.12 W/m·K) The thickness of CNF substrate (*K*_xy_ = 1.1 W/m·K, *K*_z_ = 0.23 W/m·K)^45^ was 200 µm. The ambient temperature was set to 20 °C.

## Supplementary information

Supplementary Information

## Data Availability

The experiment data that support the findings of this study are available from the corresponding author upon reasonable request.

## References

[1] Reuss RH (2005). Macroelectronics: perspectives on technology and applications. Proc. IEEE.

[2] Moussessian A, Chen C, Edelstein W, Madsen S, Rosen P (2005). System concepts and technologies for high orbit SAR. IEEE MTT-S Int. Microw. Symp . Dig..

[3] Yuan H-C, Qin G, Celler GK, Ma Z (2009). Bendable high-frequency microwave switches formed with single-crystal silicon nanomembranes on plastic substrates. Appl. Phys. Lett..

[4] Seo JH (2016). Fast flexible transistors with a nanotrench structure. Sci. Rep..

[5] Jung YH (2016). A compact parylene-coated WLAN flexible antenna for implantable electronics. IEEE Antennas Wirel. Propag. Lett..

[6] Cho SJ, Jung YH, Ma Z (2015). X-band compatible flexible microwave inductors and capacitors on plastic substrate. IEEE J. Electron Devices Soc..

[7] Cook, B. S. et al. Inkjet-printed, vertically-integrated, high-performance inductors and transformers on flexible LCP substrate. In *IEEE Int. Microwave Symp*osium 1–4 (IEEE, Tampa, FL, 2014).

[8] Sun L (2010). Flexible high-frequency microwave inductors and capacitors integrated on a polyethylene terephthalate substrate. Appl. Phys. Lett..

[9] Cook BS, Cooper JR, Tentzeris MM (2013). Multi-layer RF capacitors on flexible substrates utilizing inkjet printed dielectric polymers. IEEE Microw. Wirel. Compon. Lett..

[10] Jung YH (2015). High-performance green flexible electronics based on biodegradable cellulose nanofibril paper. Nat. Commun..

[11] Inui T, Koga H, Nogi M, Komoda N, Suganuma K (2015). A miniaturized flexible antenna printed on a high dielectric constant nanopaper composite. Adv. Mater..

[12] Wang C (2012). Self-aligned, extremely high frequency III-V metal-oxide-semiconductor field-effect transistors on rigid and flexible substrates. Nano Lett..

[13] Chang, T. H. et al. High power fast flexible electronics: Transparent RF AlGaN/GaN HEMTs on plastic substrates. In *IEEE Int. Microwave Symposium* 1–4 (IEEE, Phoenix, AZ, 2015).

[14] Mhedhbi S (2016). First power performance demonstration of flexible AlGaN/GaN high electron mobility transistor. IEEE Electron Device Lett..

[15] Yeh CH (2014). Gigahertz flexible graphene transistors for microwave integrated circuits. ACS Nano.

[16] Cheng R (2014). Few-layer molybdenum disulfide transistors and circuits for high-speed flexible electronics. Nat. Commun..

[17] Jung, Y. H. et al. Releasable high-performance GaAs Schottky diodes for gigahertz operation of flexible bridge rectifier. *Adv. Electron. Mater*. **5**, 1800772 (2019).

[18] Zhang X (2019). Two-dimensional MoS 2 -enabled flexible rectenna for Wi-Fi-band wireless energy harvesting. Nature.

[19] Scarpello ML (2011). Design of an implantable slot dipole conformal flexible antenna for biomedical applications. IEEE Trans. Antennas Propag..

[20] Jiang Y (2018). Flexible film bulk acoustic wave filters toward radiofrequency wireless communication. Small.

[21] Sharifi, H. et al. First demonstration of W-band millimeter-wave flexible electronics. In *IEEE Int. Microwave Symposium* 1–4 (IEEE, Seattle, WA, 2013).

[22] Özbek, S., Grözing, M., Alavi, G., Burghartz, J. N. & Berroth, M. Three-path SiGe BiCMOS LNA on thinned silicon substrate for IoT applications. in *48th Eur. Microwave Conference* 1241–1244 (IEEE, Madrid, 2018).

[23] Özbek S (2018). 3-Path SiGe BiCMOS power amplifier on thinned substrate for IoT applications. Integration.

[24] Stapper CH, Rosner RJ (1995). Integrated circuit yield management and yield analysis: development and implementation. IEEE Trans. Semicond. Manuf..

[25] Ramesh Babu B, Parande AK, Ahmed Basha C (2007). Electrical and electronic waste: a global environmental problem. Waste Manag. Res..

[26] Webb DR, Wilson SE, Carter DE (1986). Comparative pulmonary toxicity of gallium arsenide, gallium(III) oxide, or arsenic(III) oxide intratracheally instilled into rats. Toxicol. Appl. Pharmacol..

[27] North EJ, Halden RU (2013). Plastics and environmental health: the road ahead. Rev. Environ. Health.

[28] Mishra UK, Shen L, Kazior TE, Wu Y (2008). GaN-based RF power devices and amplifiers. Proc. IEEE.

[29] Jewett SA, Makowski MS, Andrews B, Manfra MJ, Ivanisevic A (2012). Gallium nitride is biocompatible and non-toxic before and after functionalization with peptides. Acta Biomater..

[30] Cucchiella F, D’Adamo I, Gastaldi M (2017). Sustainable waste management: Waste to energy plant as an alternative to landfill. Energy Convers. Manag..

[31] Sharifi, H. et al. Microwave and millimeter-wave flexible electronics. In *IEEE Int. Microwave Symposium* 1–3 (Tampa, FL, 2014).

[32] Hussain AM, Hussain MM (2016). CMOS-technology-enabled flexible and stretchable electronics for internet of everything applications. Adv. Mater..

[33] Shahrjerdi D, Bedell SW (2013). Extremely flexible nanoscale ultrathin body silicon integrated circuits on plastic. Nano Lett..

[34] Burghartz, J. N., Harendt, C., Hoang, T., Kiss, A. & Zimmermann, M. Ultra-thin chip fabrication for next-generation silicon processes. In *IEEE Bipolar/BiCMOS Circuits and Technology Meeting* 131–137 (Capri, Italy, 2009).

[35] Sevilla GAT (2014). Flexible and transparent silicon-on-polymer based sub-20 nm non-planar 3D FinFET for brain-architecture inspired computation. Adv. Mater..

[36] Hwang GT (2013). In vivo silicon-based flexible radio frequency integrated circuits monolithically encapsulated with biocompatible liquid crystal polymers. ACS Nano.

[37] Navaraj WT, Gupta S, Lorenzelli L, Dahiya R (2018). Wafer scale transfer of ultrathin silicon chips on flexible substrates for high performance bendable systems. Adv. Electron. Mater..

[38] Landesberger, C., Klink, G., Schwinn, G. & Aschenbrenner, R. New dicing and thinning concept improves mechanical reliability of ultra thin silicon. In *Proc. Int. Symposium on Advanced Packaging Materials Processes, Properties and Interfaces* 92–97 (Braselton, GA, 2001).

[39] Meitl MA (2006). Transfer printing by kinetic control of adhesion to an elastomeric stamp. Nat. Mater..

[40] Carlson A, Bowen AM, Huang Y, Nuzzo RG, Rogers JA (2012). Transfer printing techniques for materials assembly and micro/nanodevice fabrication. Adv. Mater..

[41] Bower CA (2017). Emissive displays with transfer-printed assemblies of 8 μm × 15 μm inorganic light-emitting diodes. Photonics Res..

[42] Radauscher EJ (2017). Miniaturized LEDs for flat-panel displays. Proc. SPIE, Light-Emitting Diodes: Mater., Devices, Appl. Solid State Lighting XXI.

[43] EpiGan. Available at: https://www.epigan.com/nl/products/rf-26.html. Accessed 2019.

[44] IEEE Standard for Information technology-Telecommunications and information exchange between systems Local and metropolitan area networks-Specific requirements - Part 11: Wireless LAN Medium Access Control (MAC) and Physical Layer (PHY) Specifications, in *IEEE Std 802.11-2016 (Revision of IEEE Std 802.11-2012). *1–3534 (2016).

[45] Shimazaki Y (2007). Excellent thermal conductivity of transparent cellulose nanofiber/epoxy resin nanocomposites. Biomacromolecules.

[46] Glassbrenner CJ, Slack GA (1964). Thermal conductivity of silicon and germanium from 3°K to the melting point. Phys. Rev..

[47] Lee KJ (2006). Bendable GaN high electron mobility transistors on plastic substrates. J. Appl. Phys..

[48] Defrance N (2013). Fabrication, characterization, and physical analysis of AlGaN/GaN HEMTS on flexible substrates. IEEE Trans. Electron Devices.

[49] Zhu H (2014). Highly thermally conductive papers with percolative layered boron nitride nanosheets. ACS Nano.

[50] Oh, S. K. et al. High-power flexible AlGaN/GaN heterostructure field-effect transistors with suppression of negative differential conductance. *Appl. Phys. Lett*. **111**, 133502 (2017).

[51] Minko A (2004). AlGaN-GaN HEMTs on Si with power density performance of 1.9 W/mm at 10 GHz. IEEE Electron Device Lett..

[52] Chakraborty PS (2014). A 0.8 THz *f*_MAX_ SiGe HBT operating at 4.3 K. IEEE Electron Device Lett..

[53] Sze, S. M. & Ng, K. K. *Physics of Semiconductor Devices* (Wiley-Interscience, 2006).

[54] Mi H (2016). Characterizations of biodegradable epoxy-coated cellulose nanofibrils (CNF) thin film for flexible microwave applications. Cellulose.

[55] Yao K (2015). Piezoelectricity-induced schottky barrier height variations in AlGaN/GaN high electron mobility transistors. IEEE Electron Device Lett..

[56] Blanton, E. W., Siegel, G., Prusnick, T. A., Glavin, N. R. & Snure, M. Strain-induced changes in AlGaN/GaN two-dimensional electron gas structures with low surface state densities. *Appl. Phys. Lett*. **113**, 263503 (2018).

[57] Lesecq M (2011). High performance of AlGaN/GaN HEMTs reported on adhesive flexible tape. IEEE Electron Device Lett..

[58] Glavin NR (2017). Flexible gallium nitride for high-performance, strainable radio-frequency devices. Adv. Mater..

[59] Isaak, R. et al. The first 0.2 μm 6-inch GaN-on-SiC MMIC process. In *Int. Conference on Compound Semiconductor Manufacturing Technology* (2014).

[60] Hongtao Xu (2004). A C-band high-dynamic range GaN HEMT low-noise amplifier. IEEE Microw. Wirel. Compon. Lett..

[61] Jiang F (2018). Wood-based nanotechnologies toward sustainability. Adv. Mater..

[62] Lien DH (2014). All-printed paper memory. ACS Nano.

[63] Lee BH (2016). Foldable and disposable memory on paper. Sci. Rep..

[64] Kumar V, Shakher C (2015). Measurement of temperature and temperature profile of candle flame using holo-shear lens and Fourier fringe analysis technique. Opt. Eng..

