# Supplementary material for: Heterogeneously integrated flexible microwave amplifiers on a cellulose nanofibril substrate
Source: Nat Commun. 2020 Jun 19;11:3118. doi: 10.1038/s41467-020-16957-4 (PMC7305312; doi:10.1038/s41467-020-16957-4)
Supplement: Supplementary file 1 — Supplementary Information [file 41467_2020_16957_MOESM1_ESM.pdf]

Supplementary Information

Heterogeneously integrated flexible microwave amplifiers on a cellulose nanofibril substrate

Zhang et al.

Supplementary Figures

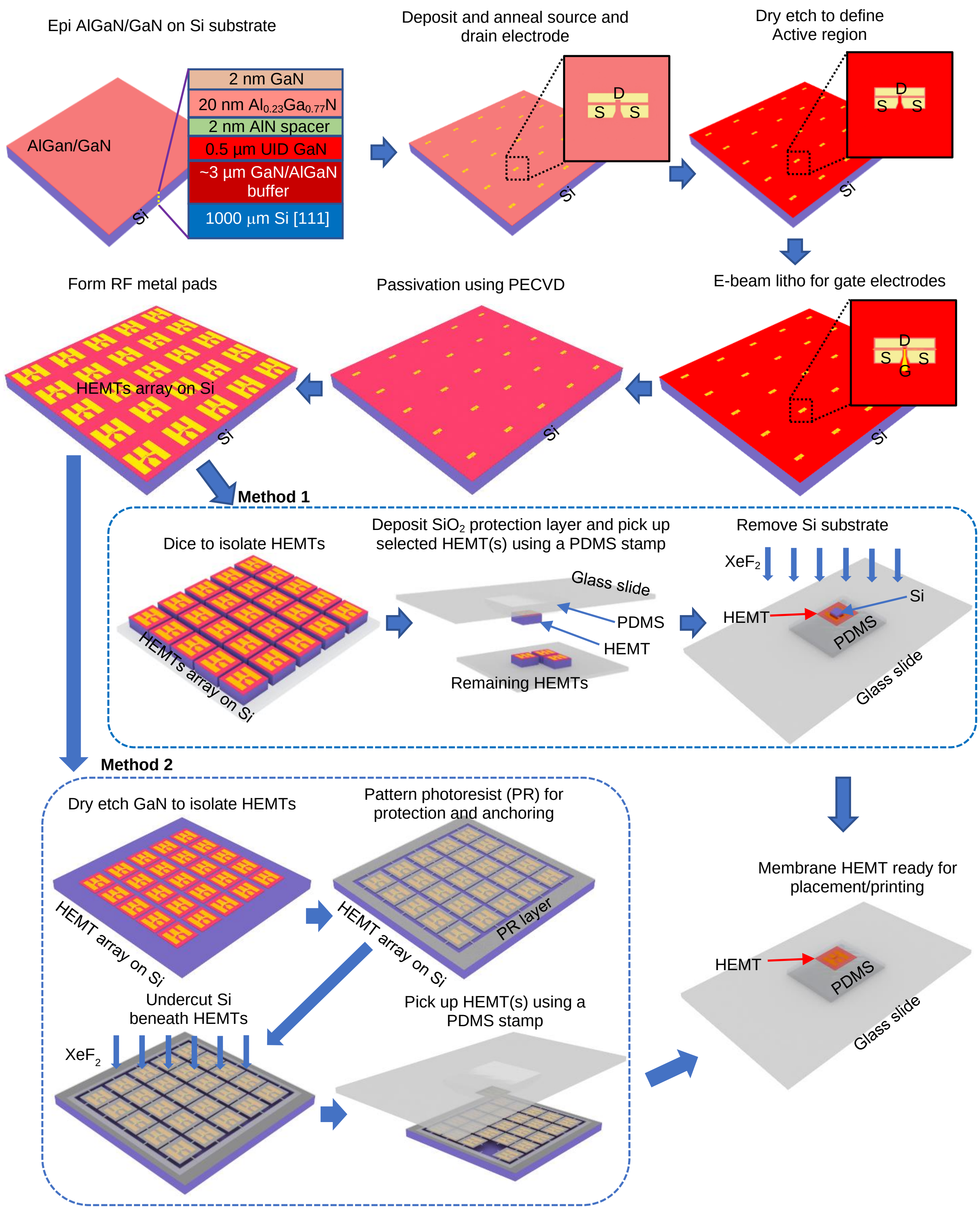

Supplementary Figure 1 | Schematic fabrication process flow of membrane AlGaN/GaN HEMTs.

Process flow of fabricating HEMT array on Si and transferring membrane HEMTs to a PDMS stamp are illustrated. The AlGaN/GaN HEMT epitaxial layers, from bottom to top, consisting of ~3 μm thick AlGaN/GaN buffer layer, a 0.5 μm unintentionally doped (UID) GaN channel layer, a 2 nm AlN spacer layer, a 20 nm $Al_{0.23}Ga_{0.77}N$ barrier layer, and a 2 nm GaN cap layer, were grown on a 1 mm thick Si (111) substrate using a metal-oxide chemical vapor deposition (MOCVD) system. A dense array of AlGaN/GaN HEMTs was fabricated on the AlGaN/GaN-on-Si wafer using conventional fabrication processes including formation of ohmic contact for source and drain electrodes, definition of active region, e-beam lithography gate electrode, passivation using PECVD, and deposition of RF pads. Two methods can be employed to remove Si substrate. Method1: The array of AlGaN/GaN HEMTs was subsequently diced into discrete AlGaN/GaN HEMTs with a size ~ 500 μm × 500 μm and a layer of SiO_2 (~ 150 nm) was deposited on the AlGaN/GaN HEMT using PECVD. A selected AlGaN/GaN HEMT(s) was picked up using a PDMS stamp. The Si substrate of HEMT was etched away using XeF_2 dry etching and membrane HEMT on PDMS stamp was obtained for further use. Method 2: The HEMTs in the array were isolated by only etching GaN layer in between HEMTs. A photoresist (PR) layer was patterned to protect and anchor the isolated HEMTs. XeF_2 etching was used to fully remove the underneath Si. Selected HEMT (or multiple HEMTs) was picked up using a patterned PDMS stamp.

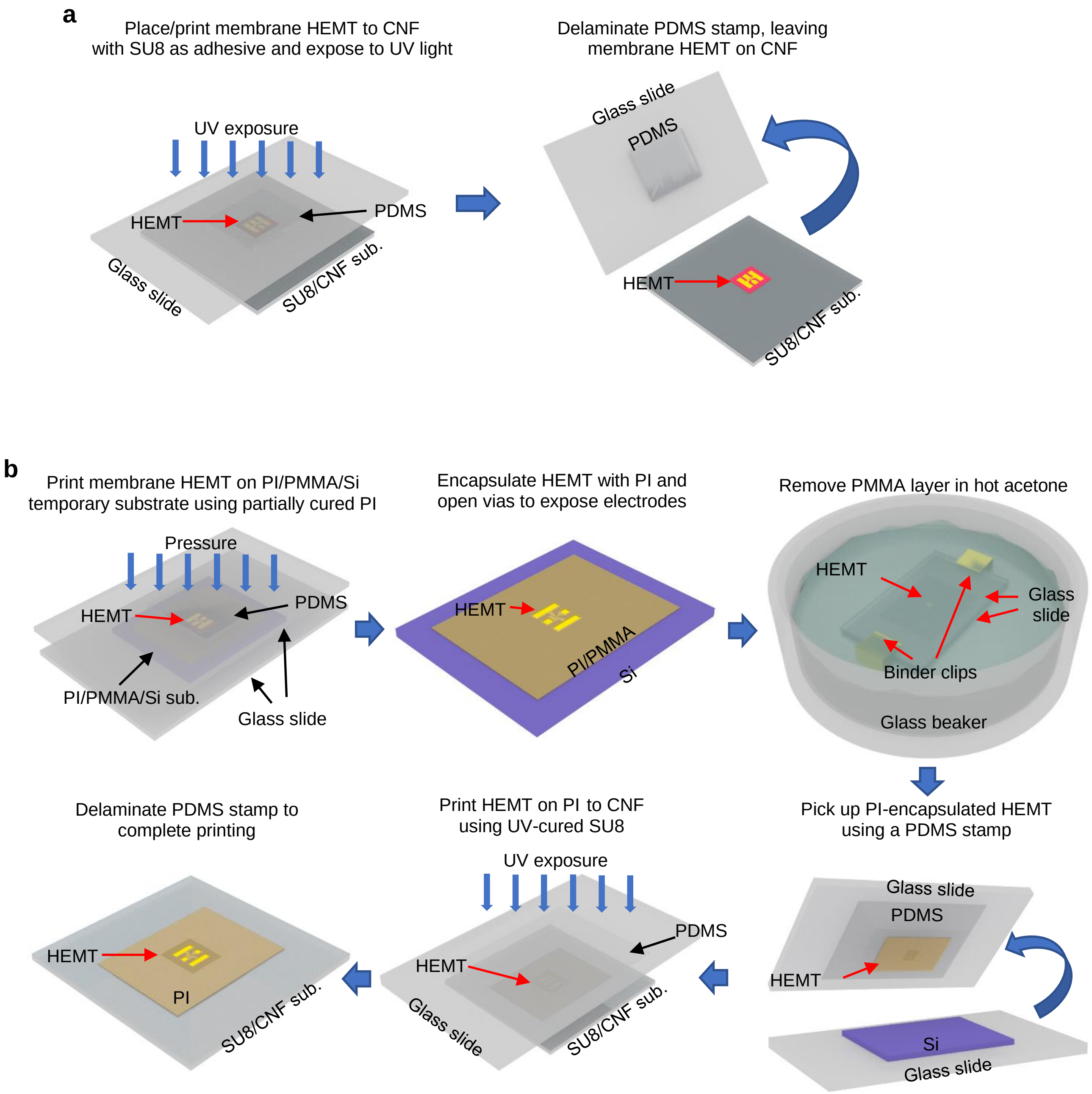

Supplementary Figure 2 | Schematic transfer-printing process of membrane AlGaN/GaN HEMTs.

a, HEMT on SU8/CNF. Process of printing membrane HEMT on CNF substrate using spin-cast SU8 as the adhesive layer is illustrated. The membrane HEMT on a PDMS stamp was attached to a CNF substrate that was spin-cast SU8 layer using a mask aligner and the sample was exposed to UV light to cure the SU8 adhesive layer. The membrane HEMT was printed on the CNF substrate after retracting the PDMS stamp. **b, HEMT on PI/SU8/CNF.** Process of printing membrane HEMT on polyimide (PI)/SU8-coated CNF substrate is illustrated. The membrane HEMT on a PDMS stamp was attached to a PI and poly (methyl

methacrylate) (PMMA)-coated Si temporary substrate with partially cured PI as adhesive layer. Another PI layer was used to encapsulate the printed HEMT. After curing PI and etching via holes on the top PI layer to expose electrodes of membrane HEMT, the PMMA sacrificial layer was dissolved in hot acetone and the membrane HEMT encapsulated by PI layers was transfer-printed to SU8-coated CNF substrate with spin-cast SU8.

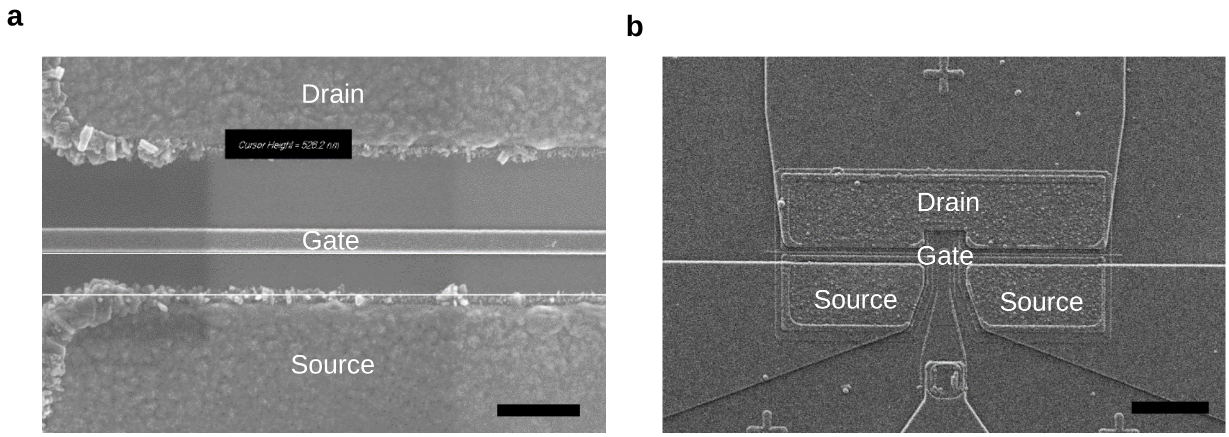

Supplementary Figure 3 | Images of the active region of AlGaN/GaN HEMT on Si.

Scanning electron microscope (SEM) images of the active region of AlGaN/GaN HEMT **a,** on Si after deposition of gate metal and **b,** after dicing. The scale bars are 1 µm and 20 µm in **a** and **b**, respectively. Due to conformal coating of the Si_3N_4 passivation layer deposited by plasma-enhanced chemical vapor deposition (PECVD), the gate width looks slightly larger than 300 nm in **b**.

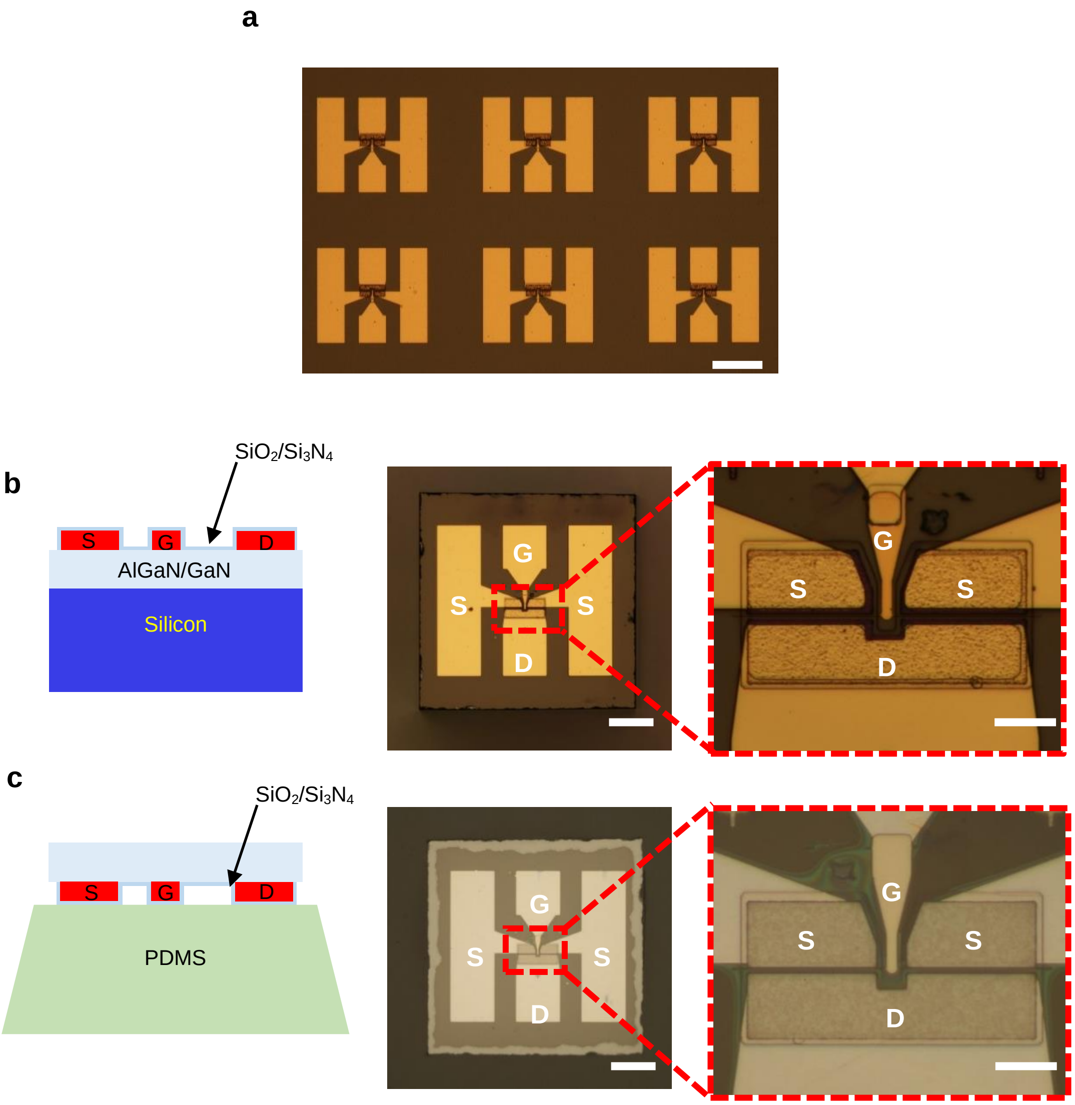

Supplementary Figure 4 | AlGaN/GaN HEMT before transfer-printing to CNF substrate.

a, An optical microscope image of part of an array of fabricated AlGaN/GaN HEMTs on Si. The scale bar is 200 µm. **b,** Cross-sectional schematic illustration (left) and optical microscope image (middle) of a diced AlGaN/GaN HEMT on Si. The magnified view shows the active region of the HEMT. **c,** Cross-sectional schematic illustration (left) and optical microscope image (middle) of membrane AlGaN/GaN HEMT sitting on a PDMS stamp. The magnified view shows the active region of the HEMT. The scale bars in the middle images and magnified views of **b** and **c** are 100 µm and 20 µm, respectively.

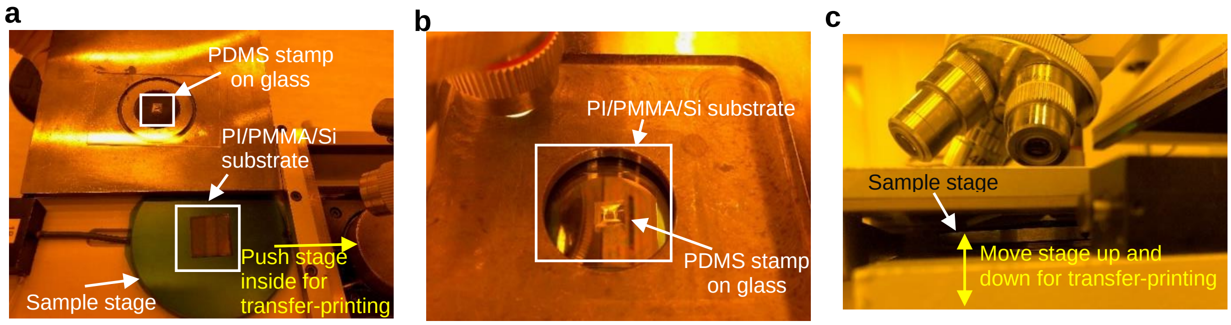

Supplementary Figure 5 | Transfer-printing setup using an MJB-3 contact aligner.

a, A PDMS stamp mounted on a glass substrate was mounted on the mask holder of the aligner. The PI/PMMA/Si substrate with partially cured polyimide adhesive layer was placed on the sample stage of the aligner. **b,** The mask holder carrying the PDMS stamp is mounted to the aligner. The sample stage with PI/PMMA/Si substrate was pushed inside the aligner's chamber. Alignment can be done at this step by adjusting the position of the sample stage. **c,** Moving the sample stage up and down for transfer-printing the membrane HEMT from the PDMS stamp to the PI/PMMA/Si substrate.

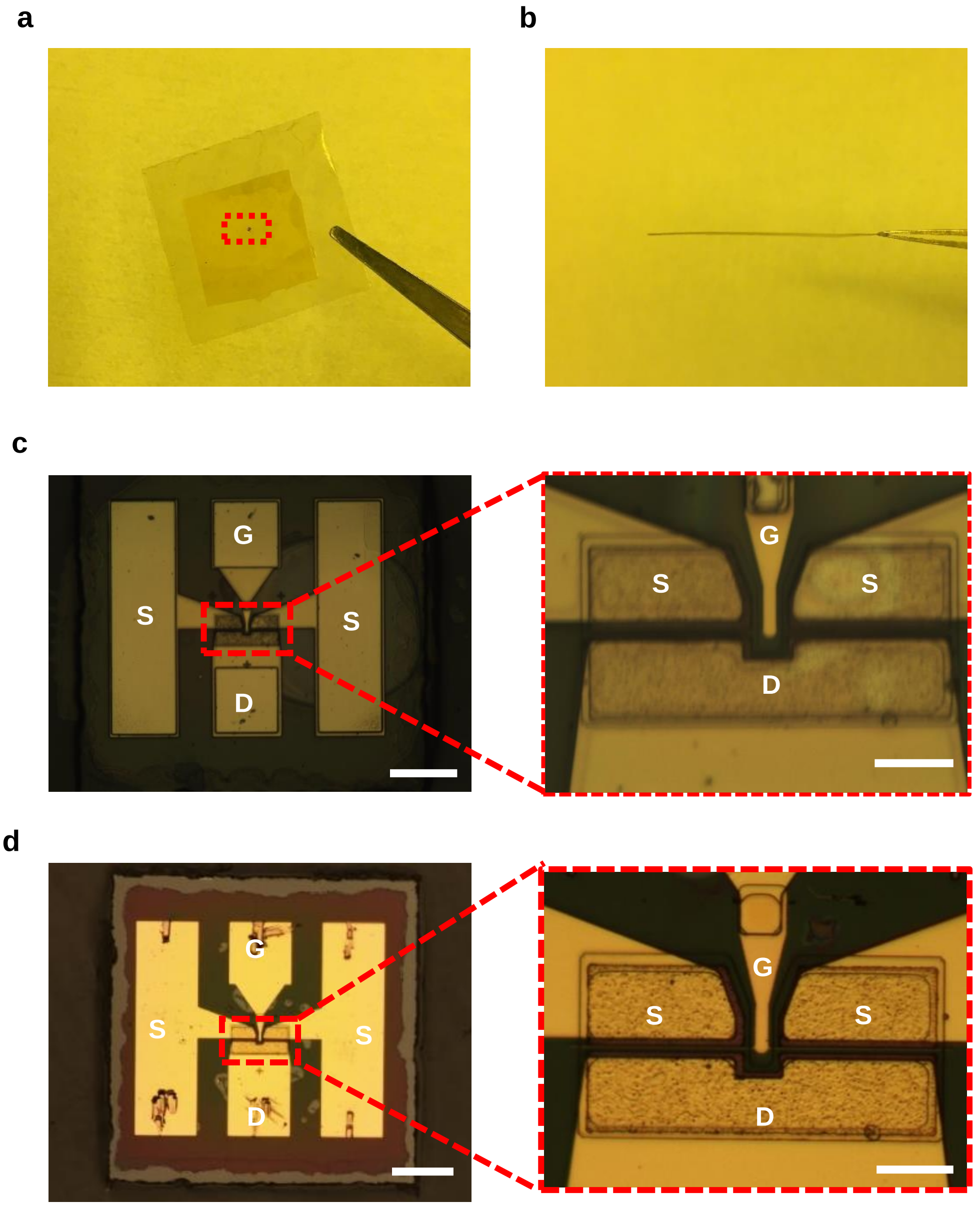

Supplementary Figure 6 | Images of flexible AlGaN/GaN HEMTs on CNF substrates.

Photographs of **a,** top-down and **b,** cross-sectional views of AlGaN/GaN HEMT on PI/SU8/CNF. The dashed red box in **a** outlines the GaN HEMT. Optical microscope images of AlGaN/GaN HEMT on **c,** PI/SU8/CNF and on **d,** SU8/CNF. The scale bars are 100 μm. Magnified views show the active regions of AlGaN/GaN HEMTs on CNF substrate. The scale bars are 20 μm. Due to DC and RF probing for characterizations, the obvious scratches are found on metal pads as in **d**.

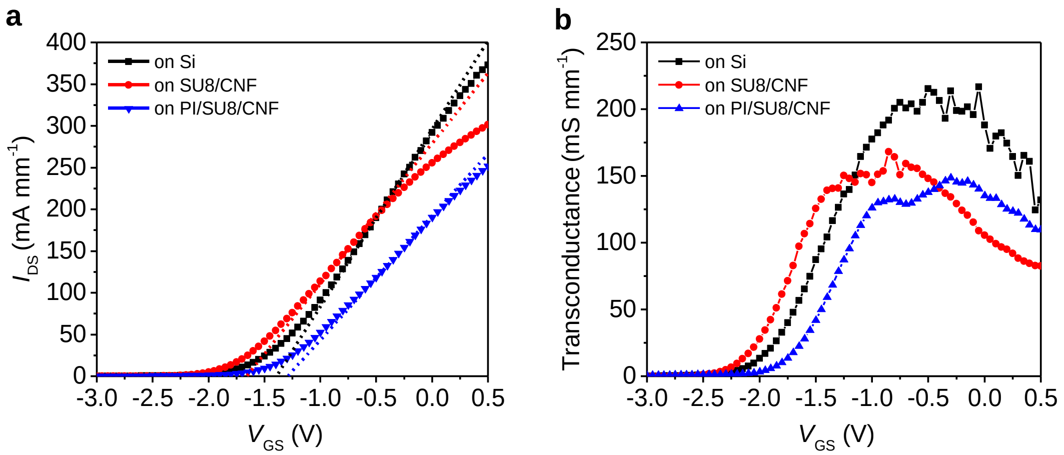

Supplementary Figure 7 | DC characteristics of discrete AlGaN/GaN HEMTs.

a, Measured drain current density and **b,** transconductance as a function of gate bias voltage (V_{GS}) of AlGaN/GaN HEMTs on Si, SU8/CNF, and PI/SU8/CNF substrates. The drain of the HEMT (V_{DS}) is biased at 10 V and the gate bias voltage (V_{GS}) is swept from -3 V to 0.5 V. Dotted lines are tangent lines of transfer curves in **a** at gate bias voltage reaching maximum transconductance. Corresponding V_{GS} for the intercept of dotted line with I_{DS} = 0 mA mm^{-1} is the threshold voltage. Note: The negative shift of threshold voltage for the HEMT sitting on SU8/CNF with reference to that sitting on Si substrate is resulted from the attacking of the HEMT active region by buffered oxide etchant (BOE, 6:1) when removing SiO_2 protection layer in order to expose metal contact pads for characterizations using the etchant[1]. The problem was eliminated when the metal pads of the HEMT was exposed using dry etching for the HEMT on PI/SU8/CNF. In this case, the threshold voltage only positively shifted by 0.09 V with reference to that of the HEMT sitting on Si substrate. The 0.09 V shift may be due to the annealing effects of the Ni/AlGaN Schottky gate of the HEMT when curing the PI[2].

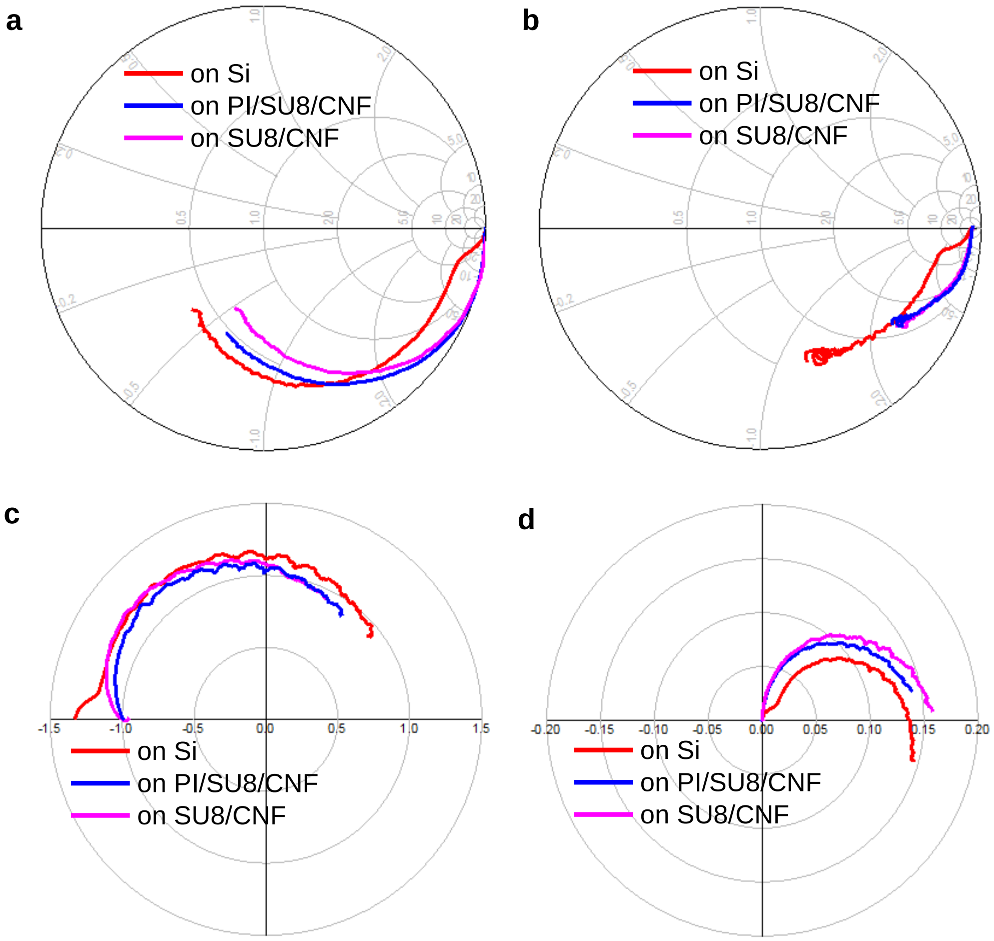

Supplementary Figure 8 | Measured S-parameters of AlGaN/GaN HEMTs.

a, S_{11} and **b,** S_{22} plotted on Smith chart for AlGaN/GaN HEMT on Si (red), SU8/CNF (pink), and PI/SU8/CNF (blue) substrates. **c,** S_{21} and **d,** S_{12} plotted on polar chart for AlGaN/GaN HEMT on Si (red), SU8/CNF (pink), and PI/SU8/CNF (blue) substrates. All HEMTs were biased at the same voltages as Fig. 3d-e, respectively.

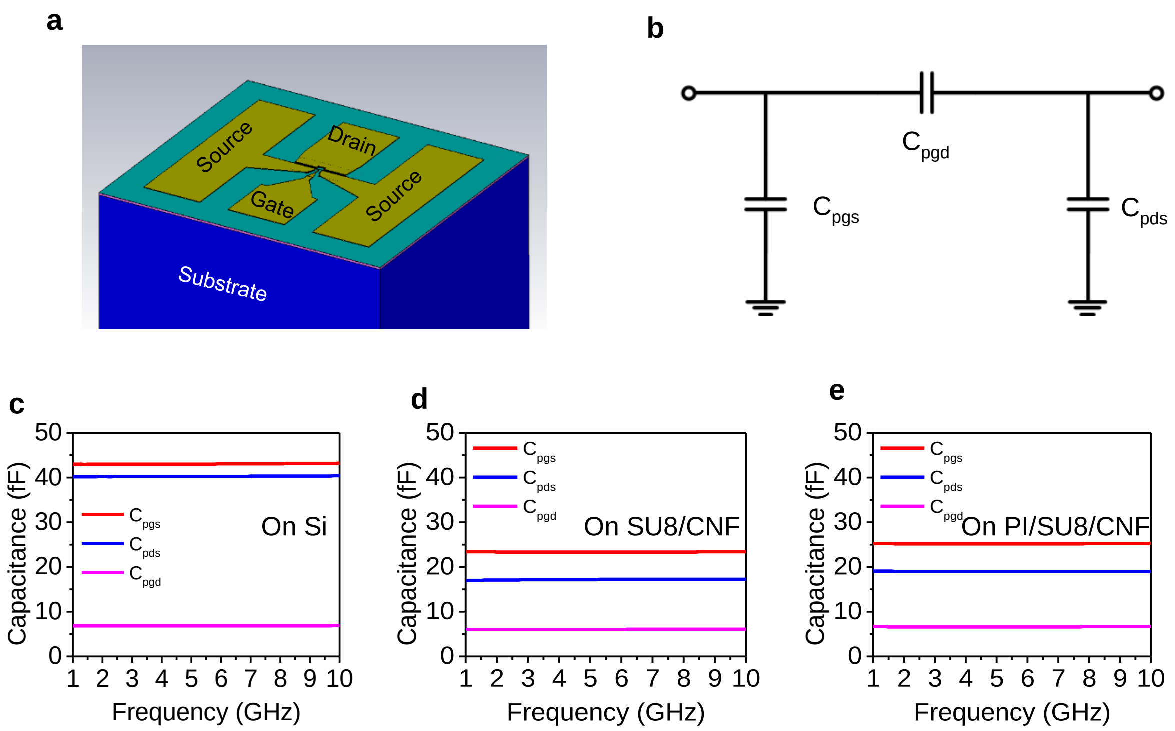

Supplementary Figure 9 | Simulations of metal pads parasitic effects on Si and CNF substrates.

a, Tilted schematic view of the simulated metal pads. The schematic cross-sectional views of the simulated structures are listed in Fig. 3a-c. **b,** Equivalent circuits of the metal pads.[3] **c-e,** Extracted parasitic capacitances from the metal pads shown in Fig. 3a-c, respectively. The extracted parasitic capacitance values of the metal pads on the three substrates at 5.5 GHz are listed in Supplementary Table 4. The metal pads on the Si substrate have significantly larger parasitic capacitances than on CNF substrate because Si has a larger dielectric constant than CNF. Due to the extra SiO_2 layer and PI encapsulation layer, the metal pads on the PI/SU8/CNF substrate have slightly larger parasitic capacitance values than that on the SU8/CNF substrate.

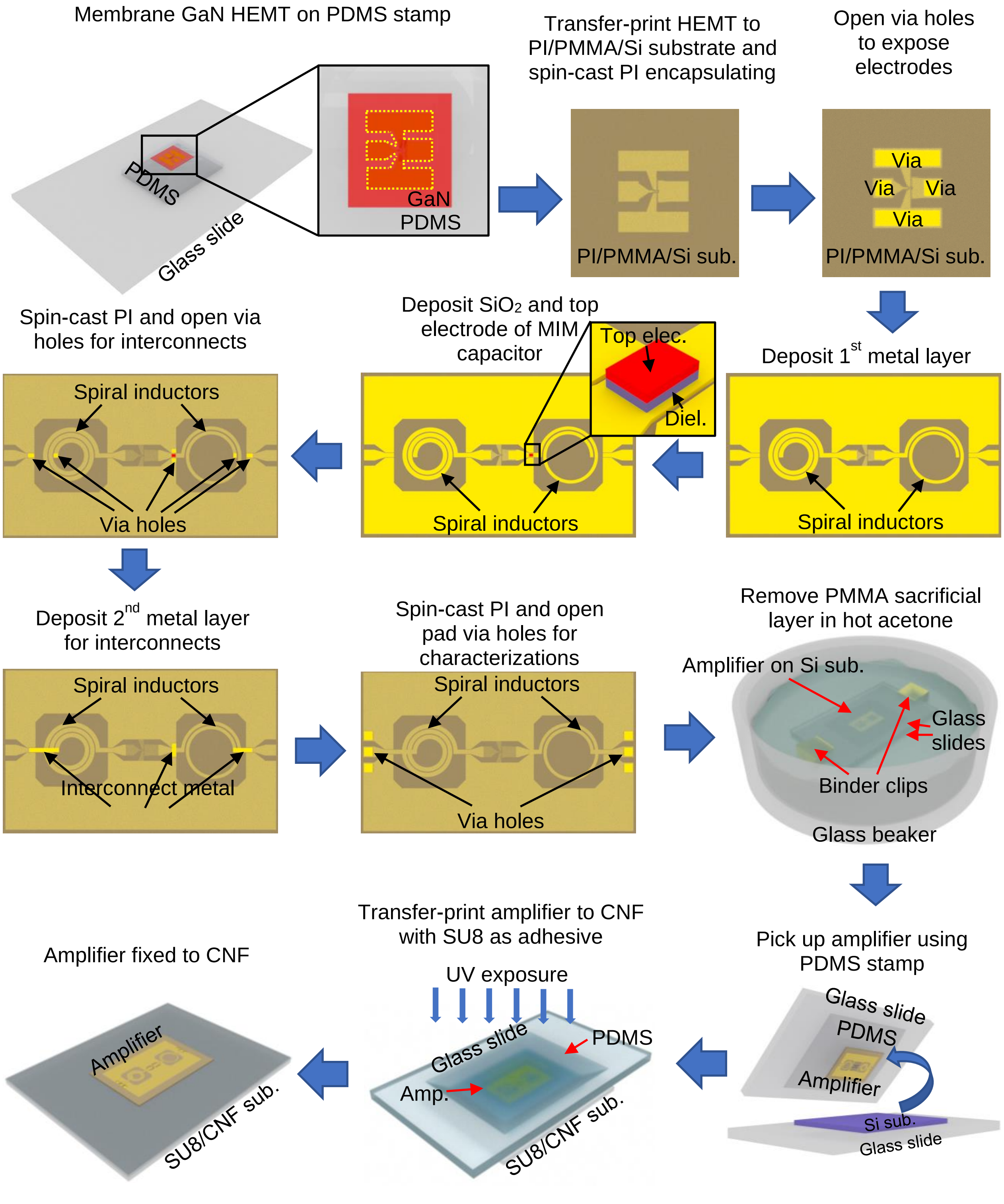

Supplementary Figure 10 | Detailed schematic process flow of flexible amplifier on CNF substrate.

The fabrication process began with transfer-printing the membrane GaN HEMT on a PDMS stamp (from the very last step of Supplementary Figure 1) to PI/PMMA/Si substrate with partially cured PI as the adhesive layer. The yellow dashed line in magnified view depicts the electrodes of GaN HEMT. A PI encapsulation layer was spin-cast on the transfer-printed HEMT and via holes were opened above metal pads of the HEMT by dry etching. The 1st metal layer, which serves as the inductor's spiral metal lines and the coplanar ground plane, was formed using contact photolithography, electron beam evaporation, and lift-off procedures. Then the capacitor's dielectric layer and the top electrode of MIM capacitor were subsequently deposited. A PI layer was spin-cast on the sample as the insulating layer. After opening via holes on the specific locations, the 2nd metal layer was deposited to form interconnects. A final encapsulating layer was spin-cast on the sample and via holes on GSG pads were opened using dry etching to expose RF pads for characterizations. The *f*MIC amplifier was picked up using a PDMS stamp after dissolving sacrificial PMMA layer in hot acetone. The amplifier, encapsulated in two PI layers, was attached to flexible CNF substrate with spin-cast SU8 as the adhesive layer and exposed to UV light for curing of the SU8 layer. After detaching the PDMS stamp from the CNF substrate, the amplifier was fixed to the flexible CNF substrate.

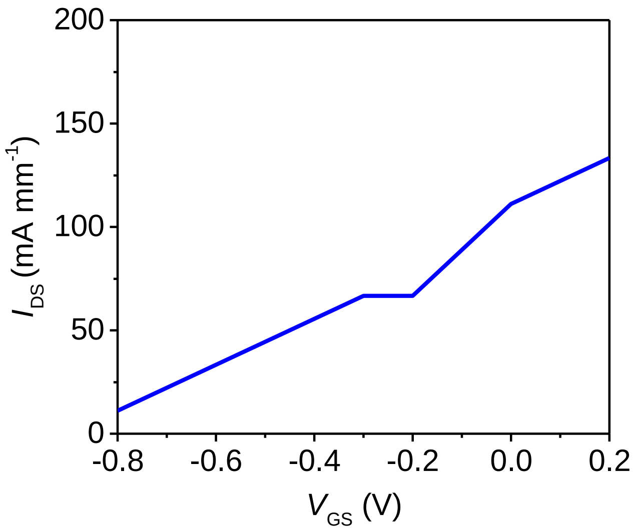

Supplementary Figure 11 | Drain current density of the HEMT in amplifier monitored as a function of gate bias voltage during small-signal RF characterizations.

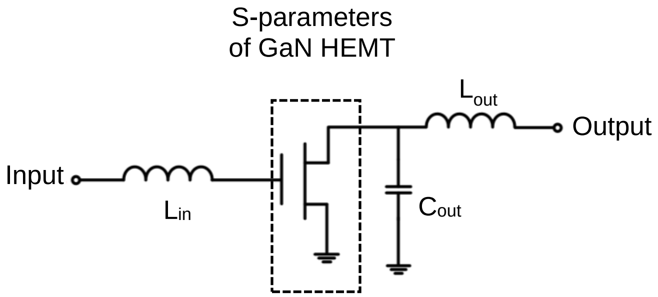

Supplementary Figure 12 | Circuit diagram for simulation of bent amplifier. (Modified from Figure 1c)

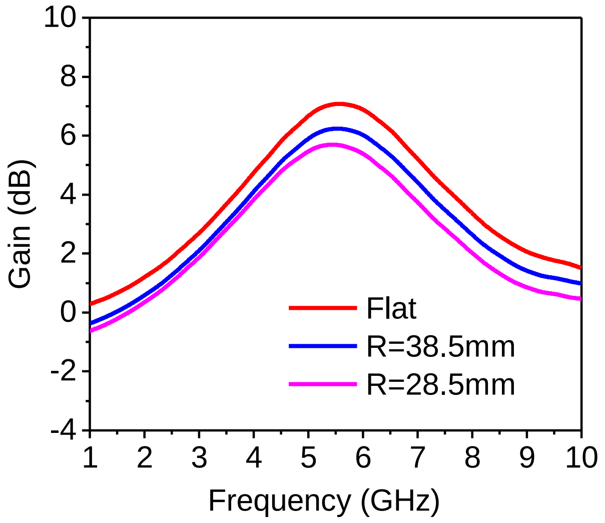

Supplementary Figure 13 | Simulated small-signal gain of the amplifier under different testing conditions.

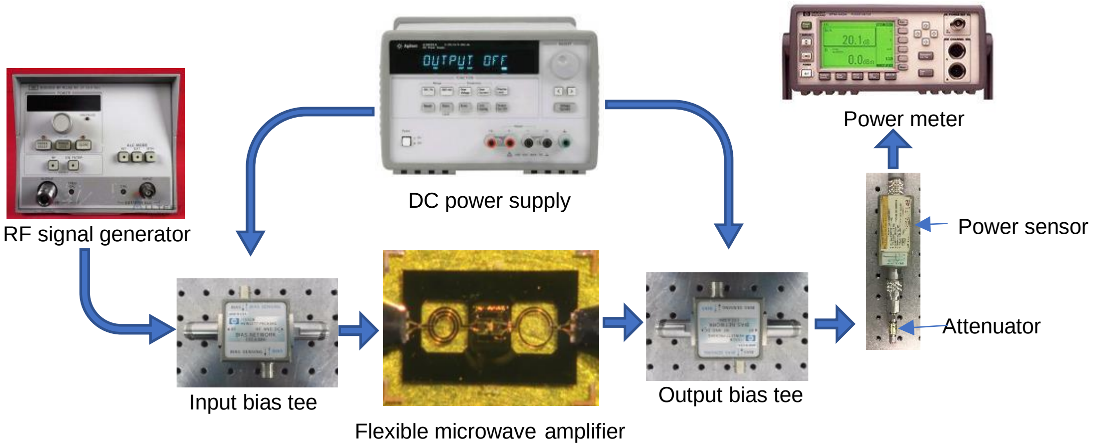

Supplementary Figure 14 | Large-signal measurement setup.

Microwave signal was generated by a microwave signal generator (HP 83592B) and combined with DC bias voltage (Agilent E3631A) through input bias tee (HP 11590B). The flexible microwave amplifier was connected to the system through microwave probe (Infinity Probe, Cascade Microtech). The output port of the flexible amplifier was connected to an output bias tee (HP 11590B), through which drain bias voltage was applied to the AlGaN/GaN HEMT. After attenuation (MOD 20600-6, Omni Spectra), output RF power was monitored by a microwave power meter (Agilent EPM-442A,) through a power sensor (HP 8481A).

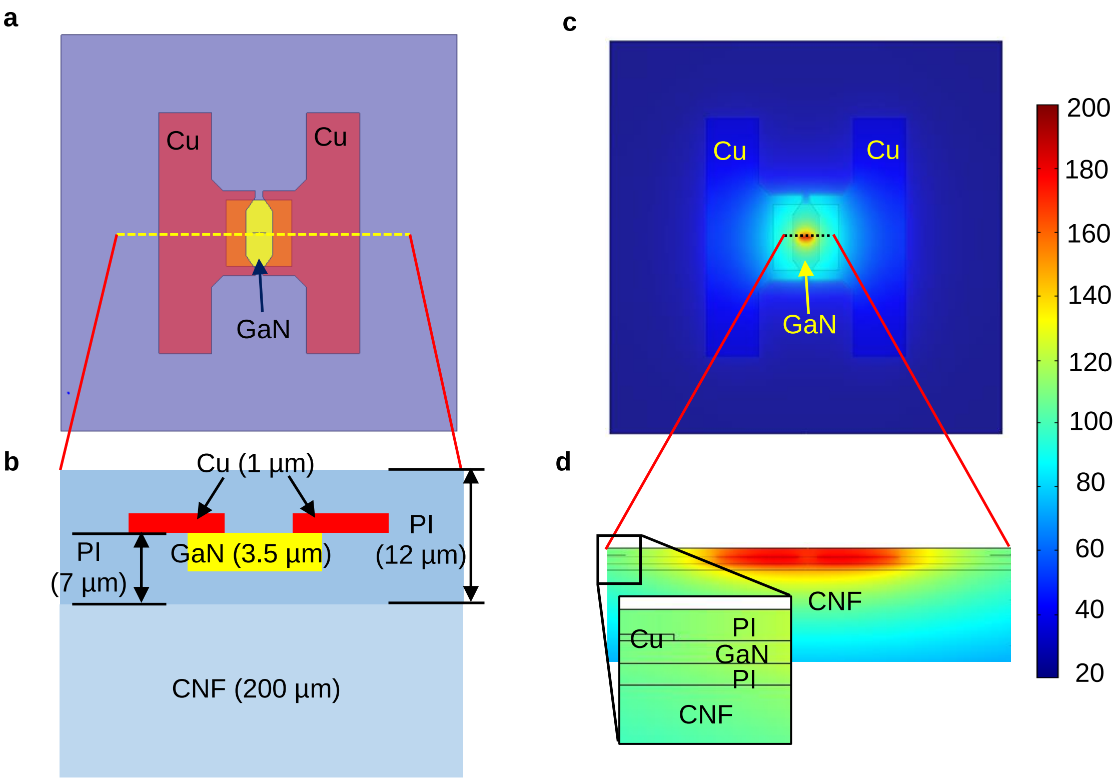

Supplementary Figure 15 | Simulations of heat dissipation in the *f*MIC.

a, Schematic layout and **b,** cross-sectional view of the device used for thermal study in COMSOL Multiphysics. In **a**, the red region represents 1 µm thick copper. The yellow region is 500 µm × 500 µm GaN membrane. **c,** Surface temperature distribution and **d,** cross-sectional temperature distribution of the simulated structure. With a DC handling power of 120 mW, *i.e.* 1.33 W/mm, the hottest spot in the circuit has a temperature of 184 °C and the CNF substrate reaches its highest temperature of 148 °C. The unit of the color scale is °C.

Supplementary Tables

Supplementary Table 1 | Comparison of MTTF of various types of microwave transistors at elevated operating temperatures

	Gate length (μm)	MTTF (hours)			Technology	Max T_C / T_J at MTTF = 1×10^6 hours (ºC)
		T_C / T_J = 200 ºC	T_C / T_J = 150 ºC	T_C / T_J = 125 ºC		
**Ref.** [4]	0.15	-	-	-	GaN HEMT (on SiC)	250
**Ref.** [5]	0.25	4.75×10^7	-	-	GaN HEMT (on SiC)	238
**Ref.** [6]	0.35	-	1.86×10^6	-	SiGe HBT	-
**Ref.** [7]	0.15	-	-	2.32×10^6	GaAs MHEMT	-
**Ref.** [8]	0.15	-	1.3×10^6	-	GaAs pHEMT	-
**Ref.** [9]	0.7	-	$> 1 \times 10^7$	-	GaN HEMT (on Si)	-

T_C/T_J: Channel temperature/Junction temperature.

Supplementary Table 2 | Threshold voltage, f_T and f_{max} values of AlGaN/GaN HEMT on different substrates.

	f_T (GHz)	f_{max} (GHz)	Threshold voltage (V)
**On Si**	34.1	69.5	-1.38
**On SU8/CNF**	37.9	87.8	-1.66
**On PI/SU8/CNF**	37.0	75.0	-1.29

Supplementary Table 3 | RF performance of reported flexible GaN HEMTs

	Gate length	f_T on rigid substrate (GHz)	f_T on flexible substrate (GHz)	f_{max} on rigid substrate (GHz)	f_{max} on flexible substrate (GHz)
Ref. [10]	400 nm (T-gate, gate head)	50	60	105	115
Ref. [11]	0.12 µm	33	32	60	52
Ref. [12]	0.1 µm	-	38	-	75
Ref. [13]	0.17 µm	-	47	-	74
**This work**	**300 nm**	**34.1**	**37.9* / 37#**	**69.5**	**87.8* / 75#**

*: on SU8/CNF substrate. #: on PI/SU8/CNF substrate.

Supplementary Table 4 | Extracted parasitic capacitance of metal pads at 5.5 GHz.

	C_{pgs} (fF)	C_{pds} (fF)	C_{pgd} (fF)
**on Si**	43.04	40.26	6.822
**on SU8/CNF**	23.34	17.21	6.034
**on PI/SU8/CNF**	25.17	19.02	6.607

Supplementary Table 5 | Small-signal gain of flexible microwave amplifier on CNF substrate.

	Gain (dB) 5 GHz	Gain (dB) 5.5 GHz	Gain (dB) 6 GHz	Peak gain (dB)	Peak-gain Frequency (GHz)
**Simulation**	6.58	6.66	6.47	6.69	5.3
**Measurement Flat (#)**	5.23 (-26.7%)	5.5 (-23.3%)	5.44 (-21.2%)	5.51 (-23.8%)	5.62
**Measurement R = 38.5 mm (*)**	5.12 (-2.4%)	5.36 (-3.1%)	5.28 (-3.7%)	5.37 (-3.4%)	5.6
**Measurement R = 28.5 mm (*)**	5.08 (-3.3%)	5.28 (-5.1%)	5.19 (-5.7%)	5.29 (-5.1%)	5.58

#: Deviation from simulation. *: Deviation from measurement under flat condition.

Supplementary Table 6 | Calculated inductance and capacitance values used for ADS simulations.

	Flat	R=38.5mm	R=28.5mm
L_{in} (nH)	3.326	3.243	3.192
L_{out} (nH)	2.244	2.188	2.154
C_{out} (pF)	0.314	0.327	0.342

Supplementary Table 7 | Comparison of reported flexible microwave amplifiers

	Semiconductor epi material area used per circuit (mm^2)	Circuit operation frequency (GHz)	Small-signal gain (dB) Flat/Bending	P_{OUT} Flat/Bending (mW)	Technology	Semiconductor thickness in circuits (μm)	P_{OUT}/Epi Area Flat (mW/mm^2)
Ref. [14]	3.74	5.5	9.7/-	6.46/-	Substrate thinning of 0.25 μm SiGe BiCMOS MMIC	45	5.17
This work	0.25	5.5	5.51/5.37*/5.29#	10.7/9.66	*f*MIC using 0.3 μm GaN HEMT	~ 3.5	42.8

*: Under bending radius of 38.5 mm. #: Under bending radius of 28.5 mm.

Supplementary Notes

Supplementary Note 1: Rational of choosing of GaN HEMT as the active device

Thermal issue is one of the biggest issues faced by microwave flexible electronics due to high-frequency operation of the active devices (transistors), from which a certain output power is needed and static DC power dissipation can cause transistor self-heating and thus substrate heating when organic substrates with very low thermal conductivity are used. Therefore, transistors that are resistant to self-heating are preferred for these applications. Special attention also needs to be paid on the influence of the heating of transistor on the substrate. Evaluation of substrate heat dissipation to avoid possible damage to the substrate is also necessary.

Based on the above considerations, GaN-based HEMT was chosen in this work for its good thermal stability as a result of its wide bandgap and superior microwave properties[15]. It is widely accepted that mean-time-to-failure (MTTF) can be used to estimate a transistor's reliability at a specific channel/junction temperature. Supplementary Table 1 summarizes several reported microwave transistors' MTTF values at elevated channel/junction temperatures, including Si, GaAs, and GaN from industry foundries. It can be seen that GaN-based HEMT can operate for a significantly longer time than GaAs- and SiGe-based transistors at higher channel/junction temperatures, which makes GaN based transistors a better choice than GaAs or SiGe based microwave transistors for flexible microwave electronics requiring a certain output power or static DC power dissipation.

Supplementary Note 2: Elaboration of cost-effectiveness, repeatability and timeline

<u>*Cost-effectiveness:*</u>

In general, the cost of fabricating a microwave circuit consists of material cost and processing cost[16], the latter of which includes the processing cost of active transistors and passive elements. Furthermore, the processing cost for passive elements is in general lower than that of processing active transistors because passive elements have a much simpler structure and larger feature size.

The demonstrated flexible microwave integrated circuit (*f*MIC) is cost-effective if one compares *f*MIC fabrication with that of monolithic microwave integrated circuits (MMICs) made with GaN or other III-V materials. To make MMICs from these materials, entire substrates were used to carry a MMIC circuit. These substrates are much more expensive than Si substrates. Furthermore, the majority of these expensive substrates were occupied by large-area passive components, such as inductors, capacitors, and transmission

lines and only a very small fraction of the substrate real-estate was used/occupied by active transistors. When converting these III-V based MMICs into *f*MICs using substrate thin-down processes such as polishing or etching, the expensive substrates were wasted[17]. It is noted that such a conversion process from MMIC into flexible MMIC made by thinning the rigid MMIC substrates adds extra cost on top of the cost of making the rigid-chip based MMICs. Moreover, because processing large-area chips leads to low manufacturing yield[18], the cost of the thin-down approach further increases.

For the *f*MIC approach described, the wafer/epi area of GaN and III-V substrates is much more effectively used than MMIC (13 times more efficient according to Supplementary Equation 1) as we made a dense array of active transistors on these substrates. Our large-area passive components were not fabricated on native III-V substrates, but eventually reside on a CNF substrate, of which the cost is almost negligible in comparison to III-V epi wafers. On the other hand, the processing cost of fabricating passive components on the temporary Si substrate is the same as that of fabricating on the III-V rigid substrates. Finally, the processing cost of removing substrate for our dense array of active devices is essentially the same as that for thinning down the substrate of a rigid MMIC. Based on the above comparisons, the cost effectiveness of our *f*MIC approach mainly comes from much more efficiently use of III-V substrates/epi materials.

The only additional cost incurred in our approach is the cost associated with the transfer printing step used to transfer our circuits from the temporary handling substrate to the CNF. It is noted that transfer printing has already been commercialized for making large-area micro LEDs displays. It is shown[19] that the transfer printing step accounts for only a very small fraction of the total cost of fabricated micro LEDs due to the high throughput and yield[20] of the automated transfer printing equipment, even though the LED fabrication process is much easier than that of HEMTs.

It is noted that for Si-based MMIC[14], although the substrate thin-down process added extra cost to that of the rigid MMIC, the inexpensive substrate of Si in the MMIC made it a cost-effective approach in addition to the advantage of maintaining the high-level metal interconnects associated with Si-based MMIC. For fabricating Si-based MMICs, our *f*MIC approach may not be a better approach than the one demonstrated in Ref.[14].

Repeatability:

With regard to the fabrication process of *f*MIC, with the exception of the transfer-printing steps using polydimethylsiloxane (PDMS) stamps, all other processing steps were performed using conventional semiconductor processing tools. It is noted that PDMS stamp based transfer-printing process has been

adopted by some semiconductor industry proving its repeatability and high fidelity (see Supplementary Note 4). The overall process for the *f*MIC fabrication is very repeatable.

Timeline:

The fabrication methods of rigid Si-based MMIC and rigid III-V based MMIC are very different. The Si-based MMICs are featured with high integration levels (*e.g.*, CMOS, HBT, passives, etc.) along with many levels of metal interconnects, of which the fabrication may take many weeks, depending on the complexity of the chips and the integration levels. In contrast, the turn-around time of III-V based MMICs is much shorter than Si-based MMICs. Converting Si-based rigid chip MMICs into flexible ones via chip thin-down simply added a few more steps to the turn-around time of the rigid ones. For the *f*MIC approach described herein, the short turn-around time of the rigid III-V MMICs is largely inherited. The processing time for the active transistors and that for the passives are essentially the same as that for their rigid counterparts. In this *f*MIC approach, substrate removal also added extra processing time in comparison to the III-V rigid ones. Moreover, our approach added additional processing time for transfer-printing. Because of the high speed of transfer-printing, the time shared by each chip is rather short. Overall, the *f*MIC processing still maintains a relatively short timeline.

Supplementary Note 3: Considerations of GaN HEMT device size and bias voltages

With different gate lengths (L_G) and gate widths (W_G), GaN HEMT can be designed for different operation frequencies and power levels. The choices of L_G and W_G of GaN HEMT in this work were based on the following considerations. The frequency goal of the *f*MIC amplifier circuit was set for 5-6 GHz. As a result, the desired f_{max} of GaN HEMT (*i.e.*, typically 10-12 times of operation frequency in order to have sufficient device gain) ought to be in the range of ~50-70 GHz. Based on reported f_T and f_{max} of rigid GaN HEMT[21–25], we chose the gate length of 300 nm. The choice of gate width was mainly based on the consideration of the desired RF output power of the amplifier circuit and that of the device channel temperature rise tolerated by the device. We set the goal of amplifier output power at ~10 mW. Considering an estimated power-added efficiency (PAE) of 5%, the DC power that the HEMT handles should be around 120 mW. According to the calculated heating power from the above values, simulations showed that the GaN HEMT temperature (the hottest point) sitting on CNF substrate would be around 184 ^{o}C. To handle 120 mW DC power, 60 µm gate width would be sufficient if a GaN HEMT was on a rigid Si substrate[26]. Since CNF substrate has a much lower thermal conductivity than Si, we had to reduce the bias voltage down to 10 V (see below). To compensate the reduced device power due to the reduced bias, we increased the gate width to 90 µm.

GaN HEMT can sustain a very high operation voltage due to the wide bandgap of GaN. The choice of drain bias is typically determined by applications. AlGaN/GaN HEMTs with similar structure to our devices are typically biased at drain voltage beyond 10 V[27,28]. However, the CNF substrate has limited thermal conductivity. To accommodate the substrate's thermal limit, we chose to lower the bias and thus to lower the operation power in order to reduce the generation of heat from the devices. The drain bias voltage could be increased to higher values if a better heat dissipation scheme was employed.

Supplementary Note 4: PDMS-based transfer-printing techniques used in this work

Following the same principle of automated transfer-printing machines already commercially available[20] and adopted for micro LED fabrication, a contact-mode mask aligner (MJB-3, Karl Suss) was employed to carry out the transfer-printing process. As shown in Supplementary Figure 5, in our setup a PDMS stamp was placed on a glass slide and the glass slide was mounted on a mask holder via vacuum. The destination substrate (*e.g.*, PI/PMMA/Si in Supplementary Fig. 5) was mounted on the sample holder on the stage of the mask aligner. By moving up the sample holder, which is similar to contacting a photoresist-coated wafer with a photo mask in contact mode photolithography, the membrane HEMT on the PDMS stamp will be attached/transferred to the destination substrate. Due to a stronger bonding force between the membrane HEMT and the destination substrate by an adhesive layer[29,30], the HEMT is transfer-printed on the destination substrate after moving down the sample stage to detach the destination substrate from the PDMS stamp. The force applied during the transfer process can be precisely adjusted by controlling the distance and speed that the sample stage was moved up by applying the needed number of turns on the adjusting knob. A transfer yield of 90%-100% can be achieved using the contact aligner-based transfer-printing method. The occasional failure of transfer-printing is typically a result of overuse of PDMS stamps, which becomes less sticky and can be "re-conditioned" with oxygen plasma treatment or simply replaced with a new one. Overall, the demonstrated *f*MIC approach in this work can be adopted for mass production using the commercially available equipment[12] in a straightforward way.

Supplementary Note 5: Comparison between flexible GaN HEMTs

The GaN-based HEMTs fabricated and used in the amplifier circuit in this work employed a similar method to what we showed before (Ref. [10], T-gate), except that a planar gate and a much smaller area of the GaN membrane were used in this work. A T-gate can achieve low gate resistance. A larger area of intrinsic GaN membrane helped to dissipate heat in the HEMT[10]. As a result, the prior HEMT[10] showed

higher RF performance than that reported in this paper. However, T-gate is more fragile than a planar gate under mechanical bending. A much larger area of GaN membrane led to reduced use efficiency of AlGaN/GaN epi area and could also limit the radius of mechanical bending. A comparison of RF performance among published work and our flexible GaN HEMT is shown in Supplementary Table 3. As can be seen, with a comparable gate length range of 120 nm- 300 nm, our HEMT showed the highest RF performances. Despite the slightly higher RF performance, the uniqueness of this demonstration is that we used the deterministic transfer-printing method to spread a dense array of HEMTs fabricated from their original expensive host epi substrates into a very sparse array on a CNF substrate, which has substantially improved the usage of epitaxial GaN materials and thus reduced the fabrication cost of microwave amplifiers based on GaN HEMTs. Of more importance, the deterministic transfer-printing method is scalable to volume production as already demonstrated in thin-film LEDs in mini/micro-LED industry[20,31].

Supplementary Note 6: Simulations of flexible amplifier under bending

The RF performance of the flexible amplifier under bending was studied through circuit simulations using ADS. The circuit diagram shown in Supplementary Figure 12 was used for the simulation. The circuit consists of ideal inductors, a capacitor, and a HEMT. The HEMT was represented/replaced with the measured S-parameters of the flexible HEMT on PI/SU8/CNF substrate under flat condition and different mechanical deformation conditions. The optimized values of the inductors and capacitor in the matching network under flat condition were initially used. To obtain the values of the inductors and capacitor under bending conditions, empirical fitting curves of inductor/capacitors values under the same bending conditions as this study were used. The empirical fitting curves were obtained in a previous study (Figure 5 in Ref. [32].). Based on the calculations, the inductance values decrease from the flat conditions by 2.5% and 4.0% when the bending radii are 38.5 mm and 28.5 mm, respectively. Similarly, the capacitance value was calculated to increase by 4.1% and 9.0% when the bending radii are 38.5 mm and 28.5 mm, respectively. Supplementary Table 6 summarizes the inductance and capacitance values used in the ADS simulations. Supplementary Figure 13 shows the simulation results of the circuit under flat and bending conditions. The decreasing trend of the small-signal gain of the amplifier under increased mechanical bending aligns with the measurement results shown in Figure 5j, which qualitatively indicates that the slight degradation of small-signal gain of the flexible amplifier under bending was a result of degraded HEMT's RF performance and the change in values of passive components due to bending.

Supplementary Note 7: Equations

The ratio between the number of transistors made on one wafer and the number of circuits made on sanme size wafer can be calculated using equation as shown below:

$$\frac{\text{Transistor per wafer}}{\text{Circuit per wafer}} \approx \frac{A_{\text{wafer}}/A_{\text{transistor}}}{A_{\text{wafer}}/A_{\text{circuit}}} \approx \frac{A_{\text{circuit}}}{A_{\text{transistor}}} = \frac{1.4\ \text{mm} \times 2.4\ \text{mm}}{0.5\ \text{mm} \times 0.5\ \text{mm}} \approx 13 \quad (1)$$

The large-signal power gain can be calculated using equation as shown below:

$$\text{Large-signal power gain} = P_{\text{OUT}}/P_{\text{IN}} \quad (2)$$

The power-added efficiency (PAE) can be calculated using equation as shown below:

$$PAE = (P_{\text{OUT}}\text{-}P_{\text{IN}})/P_{\text{DC}} \quad (3)$$

Where, P_{OUT} is output microwave power, P_{IN} is input microwave power, P_{DC} is DC consumption power.

Supplementary References

1. Lee, K. J. *et al.* Bendable GaN high electron mobility transistors on plastic substrates. *J. Appl. Phys.* **100**, 124507 (2006).

2. Arulkumaran, S., Egawa, T., Ishikawa, H., Umeno, M. & Jimbo, T. Effects of annealing on Ti, Pd, and Ni/n-Al0.11Ga0.89N Schottky diodes. *IEEE Trans. Electron Devices* **48**, 573–580 (2001).

3. Koolen, M. C. A. M., Geelen, J. A. M. & Versleijen, M. P. J. G. An improved de-embedding technique for on-wafer high-frequency characterization. in *Proceedings of Bipolar Circuits and Technology Meeting* 188–191 (1991).

4. Campbell, C. F., Nayak, S., Kao, M. Y. & Chen, S. Design and performance of 16-40GHz GaN distributed power amplifier MMICs utilizing an advanced 0.15µm GaN process. in *IEEE MTT-S International Microwave Symposium* (2016).

5. Du, J. H. *et al.* RF performance improvement of 0.25um GaN HEMT foundry technology. in *International Conference on Compound Semiconductor Manufacturing Technology* 47–50 (2015).

6. Gaw, C., Arnold, T., Martin, R., Zhang, L. & Zupac, D. Evaluation of SiGe:C HBT intrinsic reliability using conventional and step stress methodologies. *Microelectron. Reliab.* **46**, 1272–1278 (2006).

7. Chen, S. C. *et al.* Reliability Study of 0.15um MHEMT with Vds≫3V Bias for Amplifier Application. in *Workshop - Reliability of Compound Semiconductors,* 47–63 (2007).

8. Dumka, D. C. *et al.* Development of Ka-band GaAs pHEMTs with output power over 1 W/mm. in *IEEE Compound Semiconductor Integrated Circuit Symposium,* (2010).

9. GaN Reliability Report 2018 MACOM. Available at: https://www.macom.com/gan. (Accessed: 10th February 2020)

10. Chang, T. H. *et al.* High power fast flexible electronics: Transparent RF AlGaN/GaN HEMTs on plastic substrates. in *IEEE International Microwave Symposium* 1–4 (2015).

11. Defrance, N. *et al.* Fabrication, characterization, and physical analysis of AlGaN/GaN HEMTS on flexible substrates. *IEEE Trans. Electron Devices* **60**, 1054–1059 (2013).

12. Mhedhbi, S. *et al.* First power performance demonstration of flexible AlGaN/GaN high electron mobility transistor. *IEEE Electron Device Lett.* **37**, 553–555 (2016).

13. Glavin, N. R. *et al.* Flexible gallium nitride for high-performance, strainable radio-frequency devices. *Adv. Mater.* **29**, 1701838 (2017).

14. Özbek, S. *et al.* 3-Path SiGe BiCMOS power amplifier on thinned substrate for IoT applications. *Integration* **63**, 291–298 (2018).

15. Mishra, U. K., Shen, L., Kazior, T. E. & Wu, Y. GaN-based RF power devices and amplifiers. *Proc. IEEE* **96**, 287–305 (2008).

16. Isaak, R. *et al.* The First 0.2µm 6-Inch GaN-on-SiC MMIC Process. in *International Conference on*

Compound Semiconductor Manufacturing Technology (2014).

17. Sharifi, H. *et al.* First demonstration of W-band millimeter-wave flexible electronics. in *IEEE International Microwave Symposium* 1–4 (2013).

18. Stapper, C. H. & Rosner, R. J. Integrated Circuit Yield Management and Yield Analysis: Development and Implementation. *IEEE Trans. Semicond. Manuf.* **8**, 95–102 (1995).

19. Paranjpe, A., Montgomery, J., Lee, S. M. & Morath, C. Micro-LED Displays: Key Manufacturing Challenges and Solutions. *SID Symp. Dig. Tech. Pap.* **49**, 597–600 (2018).

20. Gomez, D. *et al.* Scalability and Yield in Elastomer Stamp Micro-Transfer-Printing. in *Proceedings - Electronic Components and Technology Conference* 1779–1785 (2017).

21. Choi, P., Goswami, S., Boon, C. C., Peh, L. S. & Lee, H. S. A fully integrated 5.9GHz RF frontend in 0.25um GaN-on-SiC for vehicle-to-vehicle applications. in *IEEE Radio Frequency Integrated Circuits Symposium* 397–400 (2014).

22. Liu, B. *et al.* A Fully Integrated Class-J GaN MMIC Power Amplifier for 5-GHz WLAN 802.11ax Application. *IEEE Microw. Wirel. Components Lett.* **28**, 434–436 (2018).

23. Gustafsson, D., Leidenhed, A. & Andersson, K. Packaged 7 GHz GaN MMIC doherty power amplifier. in *IEEE Compound Semiconductor Integrated Circuit Symposium* 1–4 (2017).

24. Camarchia, V., Fang, J., Moreno Rubio, J., Pirola, M. & Quaglia, R. 7 GHz MMIC GaN doherty power amplifier with 47% efficiency at 7 dB output back-off. *IEEE Microw. Wirel. Components Lett.* **23**, 34–36 (2013).

25. Jessen, G. H. *et al.* Short-channel effect limitations on high-frequency operation of AlGaN/ GaN HEMTs for T-gate devices. *IEEE Trans. Electron Devices* **54**, 2589–2597 (2007).

26. Dumka, D. C., Lee, C., Tserng, H. Q., Saunier, P. & Kumar, M. AlGaN/GaN HEMTs on Si substrate

with 7 W/mm output power density at 10 GHz. *Electron. Lett.* **40**, 1023–1024 (2004).

27. Chumbes, E. M. *et al.* AlGaN/GaN high electron mobility transistors on Si(111) substrates. *IEEE Trans. Electron Devices* **48**, 420–426 (2001).

28. Minko, A. *et al.* AlGaN-GaN HEMTs on Si with power density performance of 1.9 W/mm at 10 GHz. *IEEE Electron Device Lett.* **25**, 453–455 (2004).

29. Ahn, J. H. *et al.* High-speed mechanically flexible single-crystal silicon thin-film transistors on plastic substrates. *IEEE Electron Device Lett.* **27**, 460–462 (2006).

30. Carlson, A., Bowen, A. M., Huang, Y., Nuzzo, R. G. & Rogers, J. A. Transfer printing techniques for materials assembly and micro/nanodevice fabrication. *Advanced Materials* **24**, 5284–5318 (2012).

31. Gomez, D. *et al.* Process Capability and Elastomer Stamp Lifetime in Micro Transfer Printing. in *Proceedings - Electronic Components and Technology Conference* 680–687 (2016).

32. Cho, S. J., Jung, Y. H. & Ma, Z. X-band compatible flexible microwave inductors and capacitors on plastic substrate. *IEEE J. Electron Devices Soc.* **3**, 435–439 (2015).
